# Supplementary material for: Multiplex, single-cell CRISPRa screening for cell type specific regulatory elements
Source: Nat Commun. 2024 Sep 18;15:8209. doi: 10.1038/s41467-024-52490-4 (PMC11411074; doi:10.1038/s41467-024-52490-4)
Supplement: Supplementary file 1 — Supplementary Information [file 41467_2024_52490_MOESM1_ESM.pdf]

Supplementary Information

Multiplex, single-cell CRISPRa screening for cell type specific regulatory elements

Florence M. Chardon[1,2†], Troy A. McDiarmid[1,2†], Nicholas F. Page[3,4,5], Riza M. Daza[1,2], Beth K. Martin[1,2], Silvia Domcke[1], Samuel G. Regalado[1], Jean-Benoît Lalanne[1], Diego Calderon[1], Xiaoyi Li[1,2], Lea M. Starita[1,6], Stephan J. Sanders[3,5,7], Nadav Ahituv[4,5,*], and Jay Shendure[1,2,6,8,9,*]

Affiliations:

1. Department of Genome Sciences, University of Washington, Seattle, WA, USA
2. Seattle Hub for Synthetic Biology, Seattle, WA, USA
3. Department of Psychiatry and Behavioral Sciences, Kavli Institute for Fundamental Neuroscience, Weill Institute for Neurosciences, University of California, San Francisco, San Francisco, CA, USA
4. Department of Bioengineering and Therapeutic Sciences, University of California, San Francisco, San Francisco, CA, USA
5. Institute for Human Genetics, University of California, San Francisco, San Francisco, CA, USA
6. Brotman Baty Institute for Precision Medicine, Seattle, WA, USA
7. Institute of Developmental and Regenerative Medicine, Department of Paediatrics, University of Oxford, Oxford, OX3 7TY, UK
8. Howard Hughes Medical Institute, Seattle, WA, USA
9. Allen Discovery Center for Cell Lineage Tracing, Seattle, WA, USA

†. These authors contributed equally to this work

* Correspondence to N.A. (Nadav.Ahituv@ucsf.edu) or J.S. (shendure@uw.edu)

Supplementary Materials

Supplementary Figures S1-S21

1. **Supplementary Fig. 1** - gRNA design pipeline, library contents, and piggyFlex gRNA delivery construct.
2. **Supplementary Fig. 2** - Functional validation of CRISPRa K562 cell lines.
3. **Supplementary Fig. 3** - Results for four independent 10x Genomics lanes from K562 screen.
4. **Supplementary Fig. 4** - Hit breakdown for screen conducted in K562 cells.
5. **Supplementary Fig. 5** - Example upregulations in K562 cells.
6. **Supplementary Fig. 6** - Inducible CRISPRa iPSC-derived neuron line functional validation, selection, and differentiation timeline.
7. **Supplementary Fig. 7** - Results for four independent 10x Genomics lanes from iPSC-derived neuron screen.
8. **Supplementary Fig. 8** - Single-cell transcriptomic characterization of iPSC-derived neurons used in screen.
9. **Supplementary Fig. 9** - Distribution of CRISPRa gRNAs in single-cell neuron transcriptome data.
10. **Supplementary Fig. 10** - Successful targeting gRNAs are enriched for genomic proximity to their paired target gene scores near target genes in the iPSC-derived neurons.
11. **Supplementary Fig. 11** - Hit breakdown for screen conducted in iPSC-derived neurons.
12. **Supplementary Fig. 12** - Comparison of K562 vs. neuronal CRISPRa screening hits.
13. **Supplementary Fig. 13** - Characteristics of gRNAs leading to upregulation at EFDR<0.1 vs. EFDR>0.1.
14. **Supplementary Fig. 14** - Epigenetic features predictive of CRISPRa hit gRNAs.
15. **Supplementary Fig. 15** - TSS and cell-type specific promoters.
16. **Supplementary Fig. 16** - Example upregulations in iPSC-derived neurons.
17. **Supplementary Fig. 17** - Cell-type specific enhancers.
18. **Supplementary Fig. 18** - SCEPTRE results for K562 cells.
19. **Supplementary Fig. 19** - SCEPTRE results for iPSC-derived neurons.
20. **Supplementary Fig. 20** - Singleton validations confirm target upregulations.
21. **Supplementary Fig. 21** - Genome-wide differential expression analyses of singleton validations in K562 cells.
22. **Supplementary Fig. 22** - Genome-wide differential expression analyses of singleton validations in iPSC-derived neurons.
23. **Supplementary Fig. 23** - Correlation of fold change estimates from singleton bulk RNA-seq and multiplex single cell CRISPRa experiments.

Supplementary information

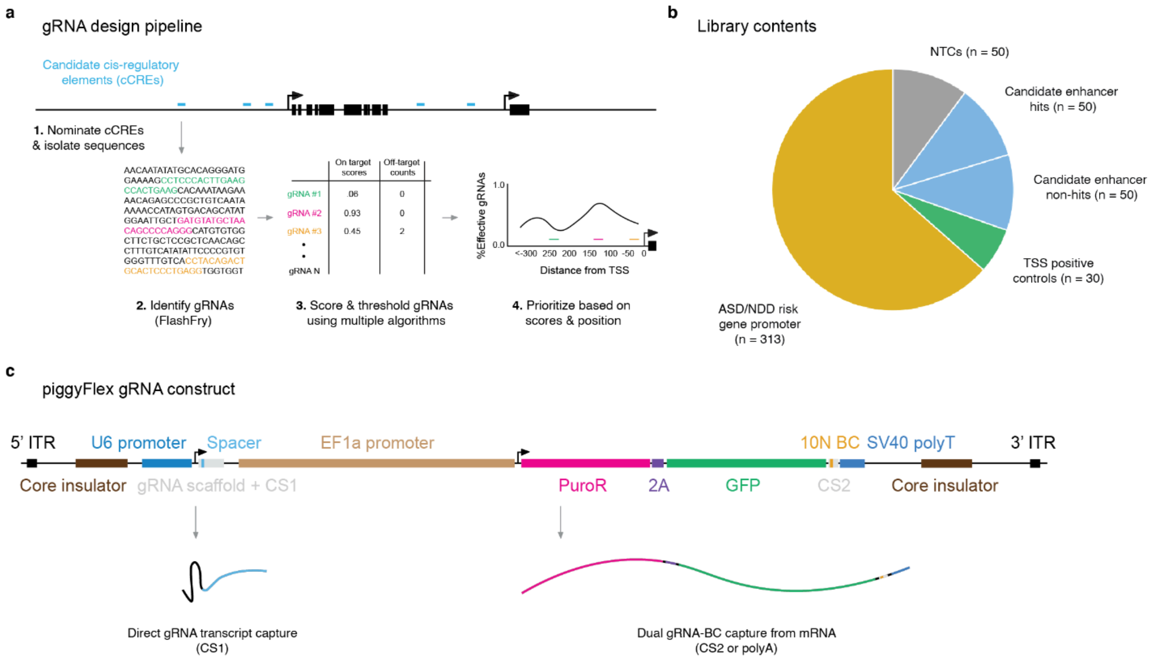

Supplementary Fig. 1 | gRNA design pipeline, library contents, and piggyFlex gRNA delivery construct. a) gRNA design pipeline. First, candidate Cis-Regulatory Elements (cCREs) surrounding a gene of interest were identified based on biochemical marks of regulatory activity (*e.g.*, accessibility, active transcription, etc.). Next, candidate gRNAs targeting each cCRE were generated using FlashFry[48]. Then, gRNAs were scored and prioritized using multiple algorithms. Finally, in the case of promoters where systematic CRISPRa design rules are available, gRNAs were prioritized based on optimal position relative to the TSS. **b)** PiggyFlex gRNA library contents by target category. **c)** PiggyFlex construct design. PiggyFlex is a piggyBac transposon-based gRNA delivery vector equipped with a dual antibiotic (puromycin) and fluorophore (GFP) selection cassette that enables enrichment for cells with many integrated gRNAs. PiggyFlex enables direct capture of gRNA transcripts or optional capture of gRNA-associated barcodes from GFP mRNA via CS2 or polydT capture.

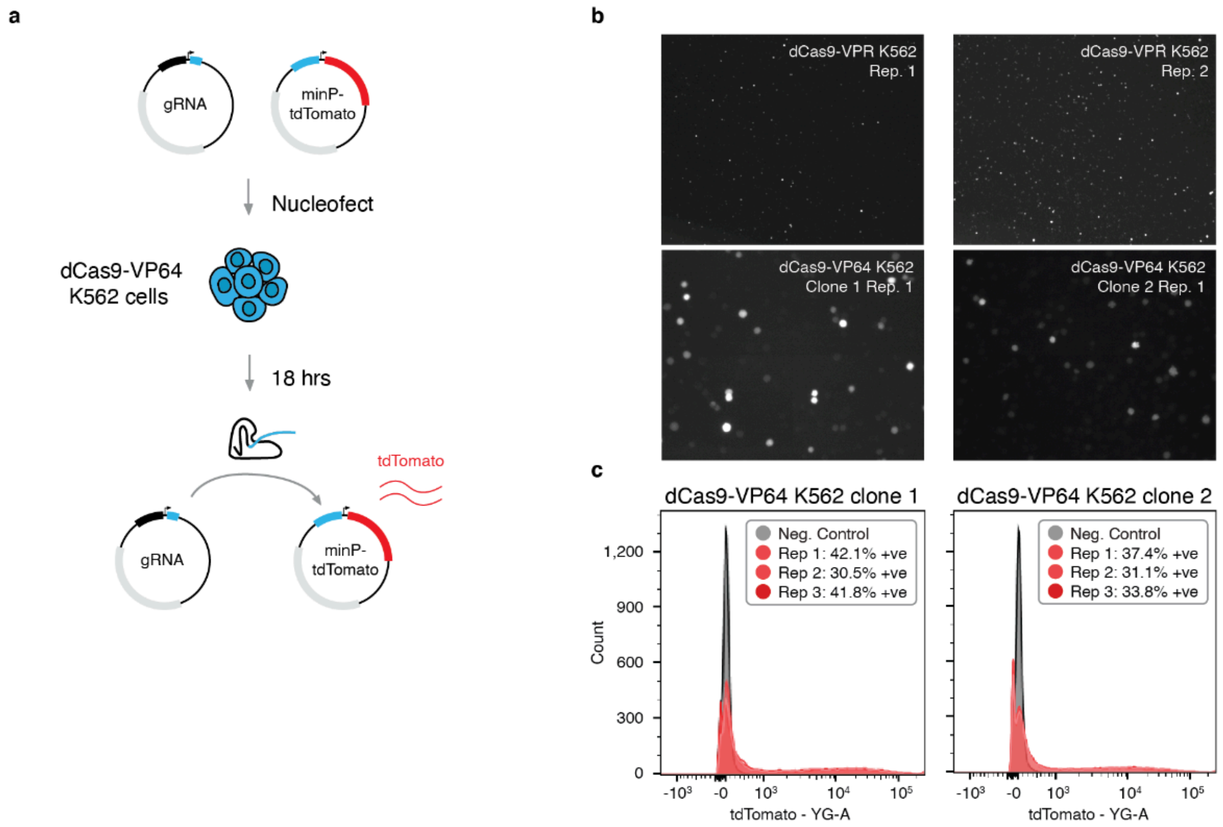

Supplementary Fig. 2 | Functional validation of CRISPRa K562 cell lines. a) Schematic of the minP-tdTomato functional assay used to validate CRISPRa cell lines. Two plasmids, one encoding a minP-tdTomato and another encoding a gRNA targeting a sequence immediately upstream of minP were co-nucleofected into K562 cell lines with either dCas9-VP64 or dCas9-VPR constructs integrated (only dCas9-VP64 is illustrated for simplicity). **b)** Following nucleofection, both dCas9-VPR (top, lower magnification) and dCas9-VP64 (bottom, higher magnification) K562 cell lines drove strong tdTomato expression, confirming the presence of functional CRISPRa machinery in these cell lines. dCas9-VPR images represent two replicate transfections into a single polyclonal line, while dCas9-VP64 images each represent one transfection replicate from two monoclonal lines. Note these are transient transfections without selection, so not all cells are expected to have been successfully transfected and fluoresced under these conditions. **c)** Example FACS analysis of tdTomato fluorescence in individual dCas9-VP64 transfection replicates of two monoclonal lines.

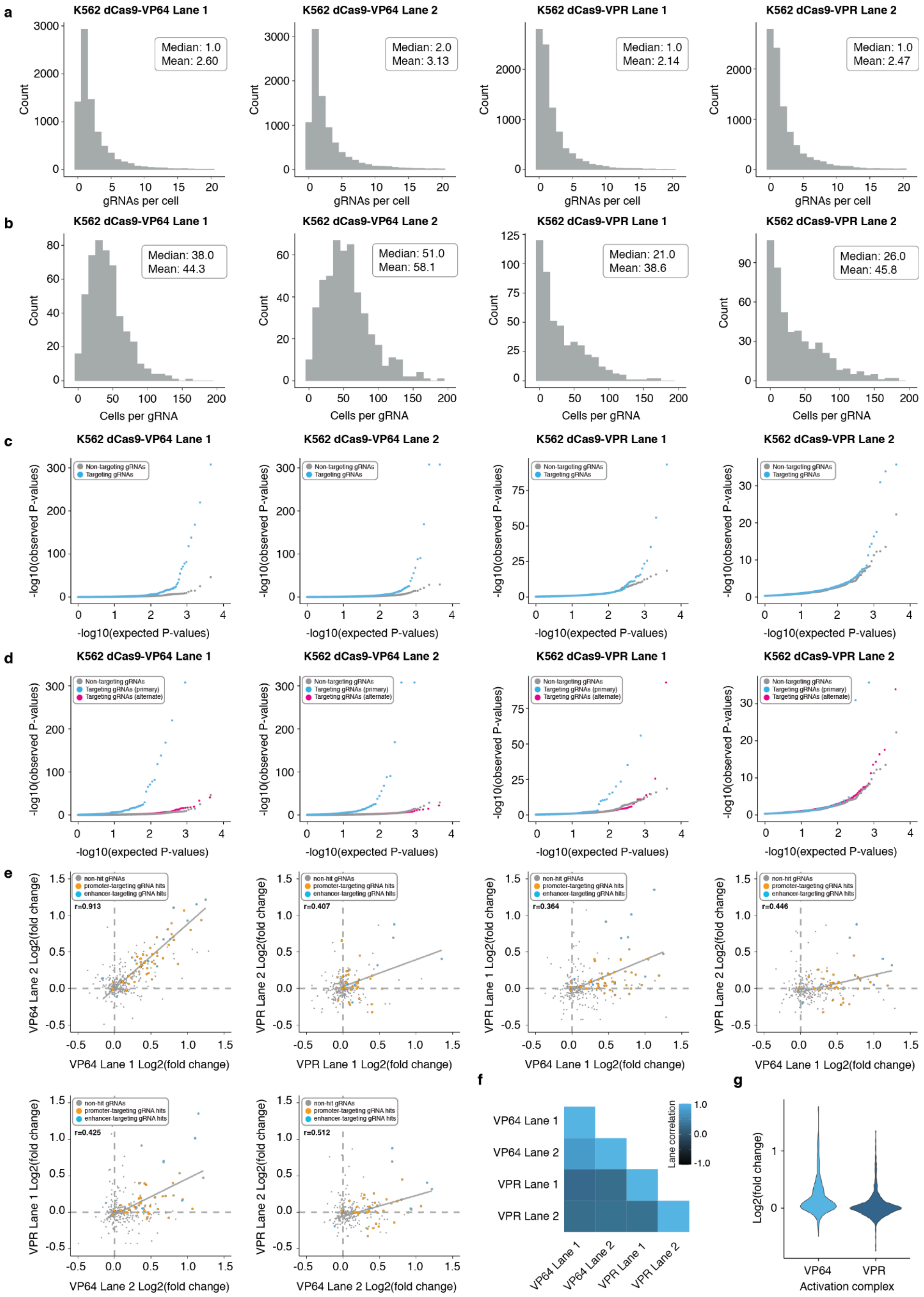

a
K562 dCas9-VP64 Lane 1
Median: 1.0
Mean: 2.60
K562 dCas9-VP64 Lane 2
Median: 2.0
Mean: 3.13
K562 dCas9-VPR Lane 1
Median: 1.0
Mean: 2.14
K562 dCas9-VPR Lane 2
Median: 1.0
Mean: 2.47
Count
gRNAs per cell
b
K562 dCas9-VP64 Lane 1
Median: 38.0
Mean: 44.3
K562 dCas9-VP64 Lane 2
Median: 51.0
Mean: 58.1
K562 dCas9-VPR Lane 1
Median: 21.0
Mean: 38.6
K562 dCas9-VPR Lane 2
Median: 26.0
Mean: 45.8
Count
Cells per gRNA
c
K562 dCas9-VP64 Lane 1
K562 dCas9-VP64 Lane 2
K562 dCas9-VPR Lane 1
K562 dCas9-VPR Lane 2
Non-targeting gRNAs
Targeting gRNAs
-log10(observed P-values)
-log10(expected P-values)
d
K562 dCas9-VP64 Lane 1
K562 dCas9-VP64 Lane 2
K562 dCas9-VPR Lane 1
K562 dCas9-VPR Lane 2
Non-targeting gRNAs
Targeting gRNAs (primary)
Targeting gRNAs (alternate)
-log10(observed P-values)
-log10(expected P-values)
e
non-hit gRNAs
promoter-targeting gRNA hits
enhancer-targeting gRNA hits
r=0.913
VP64 Lane 2 Log2(fold change)
VP64 Lane 1 Log2(fold change)
r=0.407
VPR Lane 2 Log2(fold change)
VPR Lane 1 Log2(fold change)
r=0.364
VPR Lane 1 Log2(fold change)
VP64 Lane 1 Log2(fold change)
r=0.446
VPR Lane 2 Log2(fold change)
VP64 Lane 1 Log2(fold change)
r=0.425
VPR Lane 1 Log2(fold change)
VP64 Lane 2 Log2(fold change)
r=0.512
VPR Lane 2 Log2(fold change)
VP64 Lane 2 Log2(fold change)
f
VP64 Lane 1
VP64 Lane 2
VPR Lane 1
VPR Lane 2
Lane correlation
1.0
0.0
-1.0
g
Log2(fold change)
VP64
VPR
Activation complex

Supplementary Fig. 3 | Results for four independent 10x Genomics lanes from K562 screen. a) The four 10x Genomics lanes profiled included two lanes with dCas9-VP64 K562 cells and two lanes with dCas9-VPR K562 cells. Following QC and gRNA assignment we identified an average of 2.60, 3.13, 2.14, and 2.47 gRNAs/cell for the four different 10x Genomics lanes profiled (median 2.60, 3.13, 2.14, and 2.47 gRNAs per cell). PiggyBac integrations per cell distribution is not well-modeled by a standard Poisson distribution and is better approximated by an exponential function. **b)** Multiplexing more than one perturbation per cell yielded an average of 38.0, 51.0, 21.0, and 26.0 cells/gRNA for the four different 10x Genomics lanes profiled (median 44.3, 58.1, 38.6, and 45.8 cells/gRNA). **c)** QQ-plots displaying observed vs. expected *P*-value distributions for targeting (blue) and NTC (downsampled) populations across the four different 10x Geomics lanes profiled. **d)** QQ-plots for targeting tests against their intended/programmed target (blue) compared to targeting tests of all other genes with 1Mb of each gRNA (pink) and NTCs (gray downsampled) across the four different 10x Genomics lanes profiled. **c-d)** *P*-values are from a two-tailed Wilcoxon rank-sum test. **e)** Correlation plots of log2 fold-changes of gRNAs across the two K562 cell lines (dCas9-VP64 and dCas9-VPR) for all four 10x Genomics lanes profiled. Pearson correlations of gRNA hits are shown. **f)** Matrix correlation plot displaying the Pearson correlations of the log2 fold-changes of target gene expression values for programmed targets across the four different 10x Genomics lanes profiled. **g)** Violin plot displaying the log2 fold-changes of target gene expression values for programmed targets for K562 cells harboring the dCas9-VP64 activation complex and the dCas9-VPR activation complex.

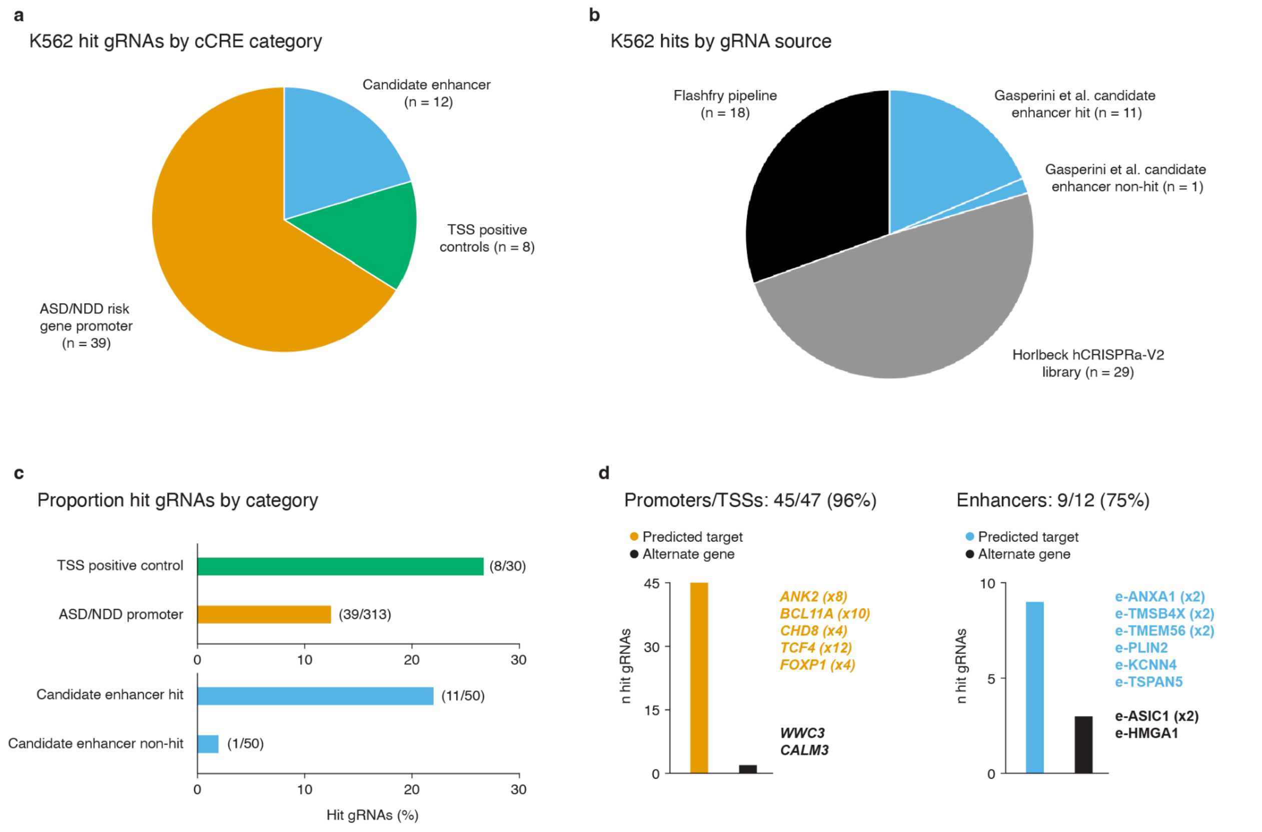

Supplementary Fig. 4 | Hit breakdown for screen conducted in K562 cells. a) K562 hit gRNAs by cCRE category. **b)** K562 hit gRNAs by gRNA source library or design pipeline. **c)** Proportion of hit gRNAs by cCRE category. **d)** Proportion of hit gRNAs yielding upregulation of their intended/expected target gene or an alternate gene for candidate promoters/TSSs (left) or enhancers (right). Example hits targeting candidate NDD risk gene promoters (left) and K562 enhancers (right) are listed. Bracketed numbers denote the number of independent hit gRNAs targeting the same cCRE.

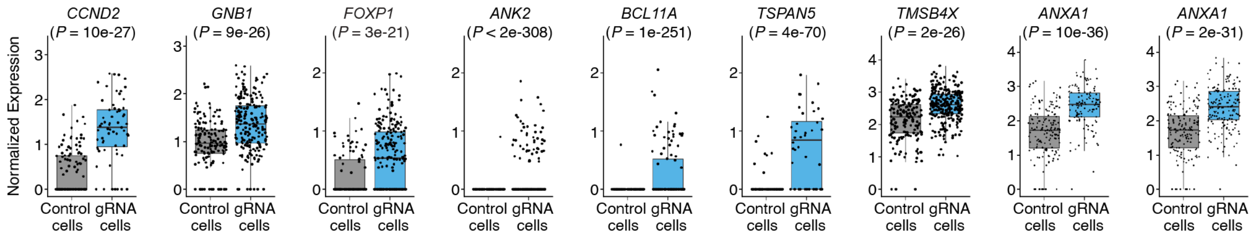

Supplementary Fig. 5 | Example upregulations in K562 cells. Example box plots showing the average log2 fold-changes between cells with a given gRNA and controls for select hit gRNAs. Hits include TSS positive controls (*CCND2*, *GNB2*), candidate promoters of genes rarely or not expressed in K562 cells (*ANK2*, *BCL11A*) and candidate K562 enhancers (*TSPAN5*, *TMSB4X*, and *ANXA1*). Number of cells bearing each targeting gRNA (from left to right): *CCND2* (n = 73), *GNB2* (n = 220), *FOXP1* (n = 313), *ANK2* (n = 403), *BCL11A* (n = 191), *TSPAN5* (n = 48), *TMSB4X* (n = 260), *ANXA1* (n = 166), *ANXA1* (n = 128). Control cells are downsampled to have the same number of cells as the average number of cells detected per gRNA (n = 178) for visualization. Boxes represent the 25th, 50th, and 75th percentiles. Whiskers extend from hinge to 1.5 times the inter-quartile range. All data points are also plotted on top of the box plot for transparency. *P*-values are from a two-tailed Wilcoxon rank-sum test. Normalized expression values represent log normalized expression values from Seurat.

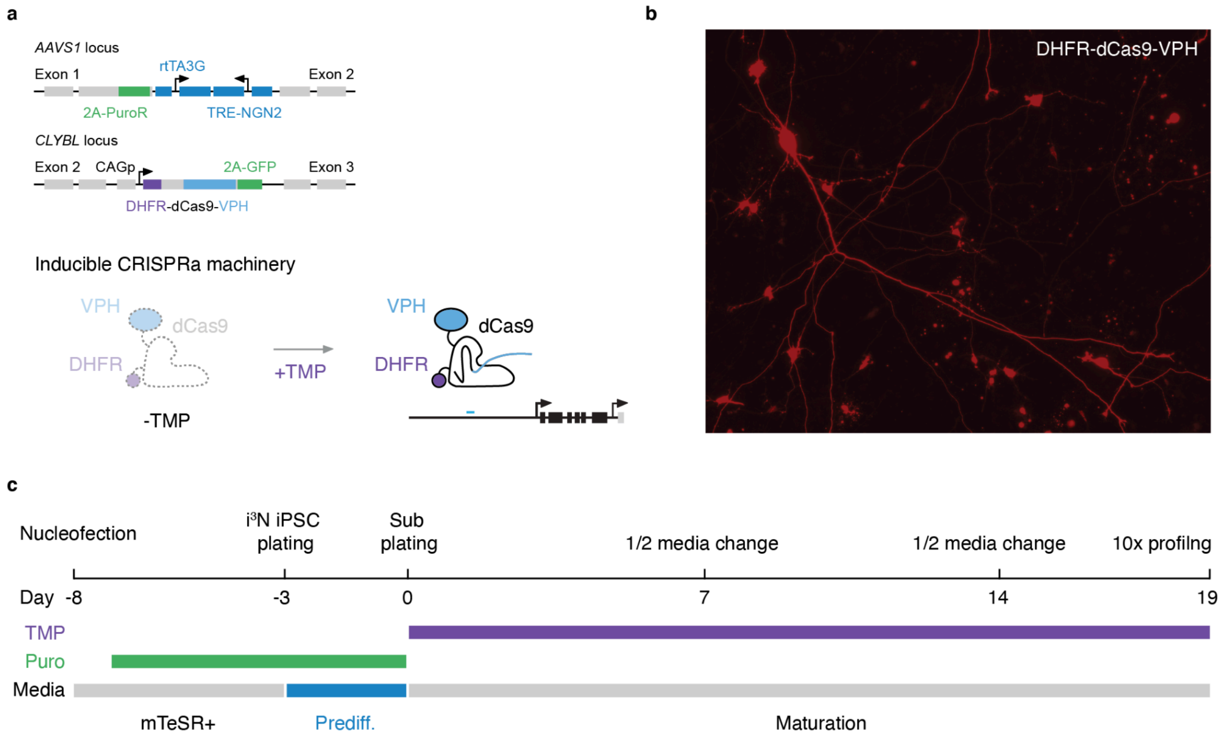

Supplementary Fig. 6 | Inducible CRISPRa iPSC-derived neuron line functional validation, selection, and differentiation timeline. a) (Top) iPSCs equipped with a Dox-inducible *NGN2* transcription factor to drive neural differentiation (integrated at the *AAVS1* safe harbor locus) and TMP-inducible CRISPRa-VPH machinery (integrated at the *CLYBL* locus) were used for all iPSC-derived neuron experiments. (Bottom) In the absence of TMP, CRISPRa-VPH machinery is degraded via a DHFR degron. In the presence of TMP, the CRISPR-VPH machinery is stabilized, enabling perturbation. **b)** Functional validation of CRISPRa machinery in iPSC-derived neurons. Neurons were lipofected with a minP-tdTomato reporter and sgRNA that targets the minimal promoter. CRISPRa machinery drove clear tdTomato expression in differentiated neurons. **c)** Nucleofection, selection, and differentiation timeline. iPSCs were nucleofected with piggyFlex gRNA constructs at a high MOI and selected with puromycin to enrich cells for with multiple integrated gRNAs. Following differentiation induction neurons were subplated in maturation media with TMP to induce CRISPRa machinery. Neurons were single cell profiled following 19 days of differentiation (10x Genomics V3.1 chemistry with direct gRNA capture).

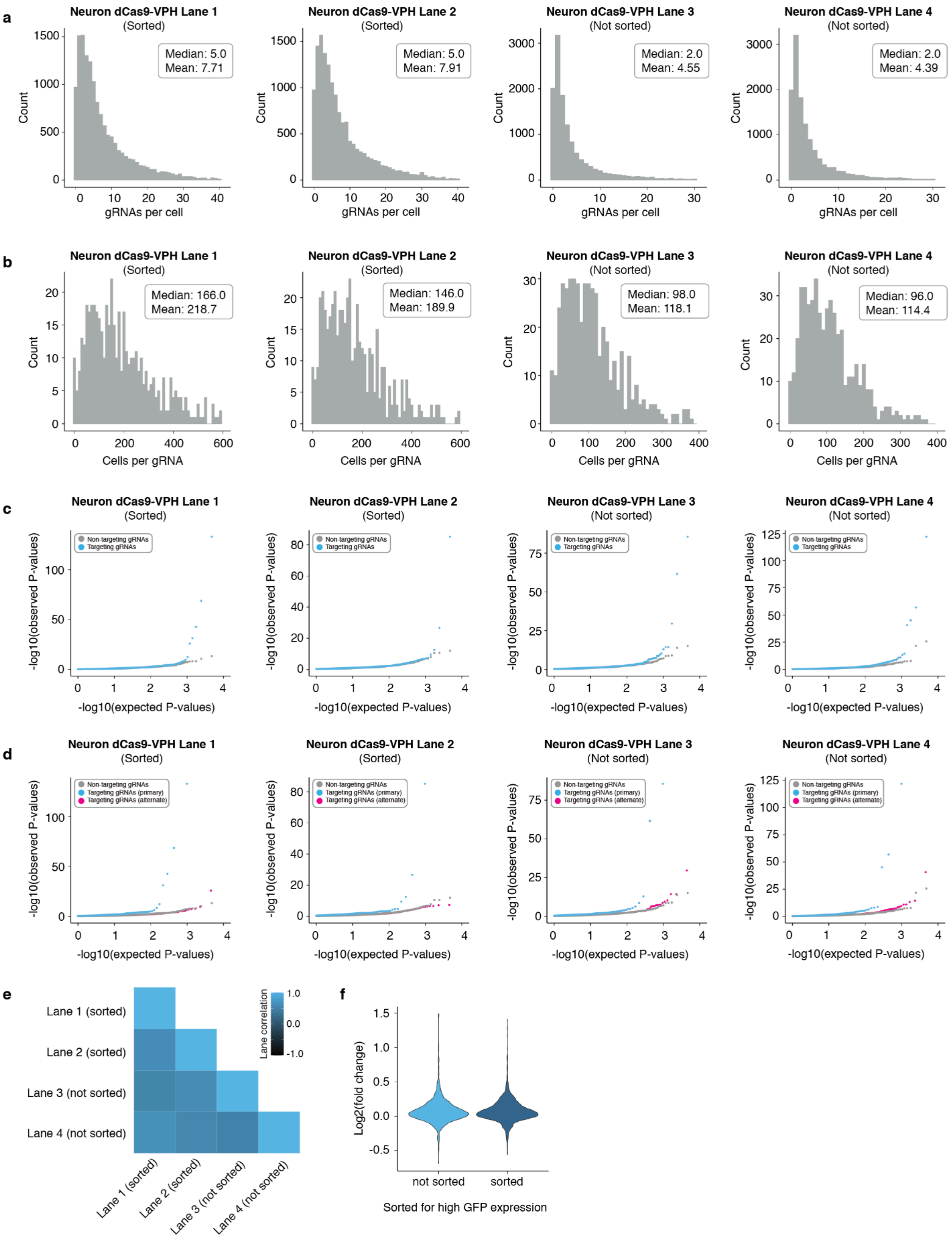

a
Neuron dCas9-VPH Lane 1 (Sorted)
Median: 5.0
Mean: 7.71
Neuron dCas9-VPH Lane 2 (Sorted)
Median: 5.0
Mean: 7.91
Neuron dCas9-VPH Lane 3 (Not sorted)
Median: 2.0
Mean: 4.55
Neuron dCas9-VPH Lane 4 (Not sorted)
Median: 2.0
Mean: 4.39
Count
gRNAs per cell
b
Neuron dCas9-VPH Lane 1 (Sorted)
Median: 166.0
Mean: 218.7
Neuron dCas9-VPH Lane 2 (Sorted)
Median: 146.0
Mean: 189.9
Neuron dCas9-VPH Lane 3 (Not sorted)
Median: 98.0
Mean: 118.1
Neuron dCas9-VPH Lane 4 (Not sorted)
Median: 96.0
Mean: 114.4
Count
Cells per gRNA
c
Neuron dCas9-VPH Lane 1 (Sorted)
Neuron dCas9-VPH Lane 2 (Sorted)
Neuron dCas9-VPH Lane 3 (Not sorted)
Neuron dCas9-VPH Lane 4 (Not sorted)
Non-targeting gRNAs
Targeting gRNAs
-log10(observed P-values)
-log10(expected P-values)
d
Neuron dCas9-VPH Lane 1 (Sorted)
Neuron dCas9-VPH Lane 2 (Sorted)
Neuron dCas9-VPH Lane 3 (Not sorted)
Neuron dCas9-VPH Lane 4 (Not sorted)
Non-targeting gRNAs
Targeting gRNAs (primary)
Targeting gRNAs (alternate)
-log10(observed P-values)
-log10(expected P-values)
e
Lane 1 (sorted)
Lane 2 (sorted)
Lane 3 (not sorted)
Lane 4 (not sorted)
Lane correlation
1.0
0.0
-1.0
f
Log2(fold change)
not sorted
sorted
Sorted for high GFP expression

Supplementary Fig. 7 | Results for four independent 10x Genomics lanes from iPSC-derived neuron screen. a) The four 10x Genomics lanes profiled consisted of two lanes with dCas9-VPH neurons that were sorted on the top 40% of GFP expression in these cells, and two lanes that were not on the top 40% of GFP expression in these cells. The cells that were not sorted were still 100% GFP+. Following QC and gRNA assignment we identified an average of 7.71, 7.91, 4.55, and 4.39 gRNAs/cell for the four different 10x Genomics lanes profiled (median 7.71, 7.91, 4.55, and 4.39 gRNAs per cell). Note that sorting neurons on the top 40% of GFP expression boosted the median and mean gRNAs/cell ~2 fold. PiggyBac integrations per cell distribution is not well-modeled by a standard Poisson distribution and is better approximated by an exponential function. **b)** Multiplexing multiple perturbations per cell yielded an average of 218.7, 189.9, 118.1, and 114.4 cells/gRNA for the four different 10x Genomics lanes profiled (median 166, 146, 98, and 96 cells/gRNA). **c)** QQ-plots displaying observed vs. expected *P*-value distributions for targeting (blue) and NTC (downsampled) populations across the four different 10x Genomics lanes profiled. **d)** QQ-plots for targeting tests against their intended/programmed target (blue) compared to targeting tests of all other genes with 1Mb of each gRNA (pink) and NTCs (gray downsampled) across the four different 10x Genomics lanes profiled. **c-d)** *P*-values are from a two-tailed Wilcoxon rank-sum test. **e)** Matrix correlation plot displaying the Pearson correlations of the log2 fold-changes of target gene expression values for programmed targets across the four different 10x Genomics lanes profiled. **f)** Violin plot displaying the log2 fold-changes of target gene expression values for programmed targets for neurons that were sorted on the top 40% GFP expression (sorted) and neurons that were not sorted (not sorted).

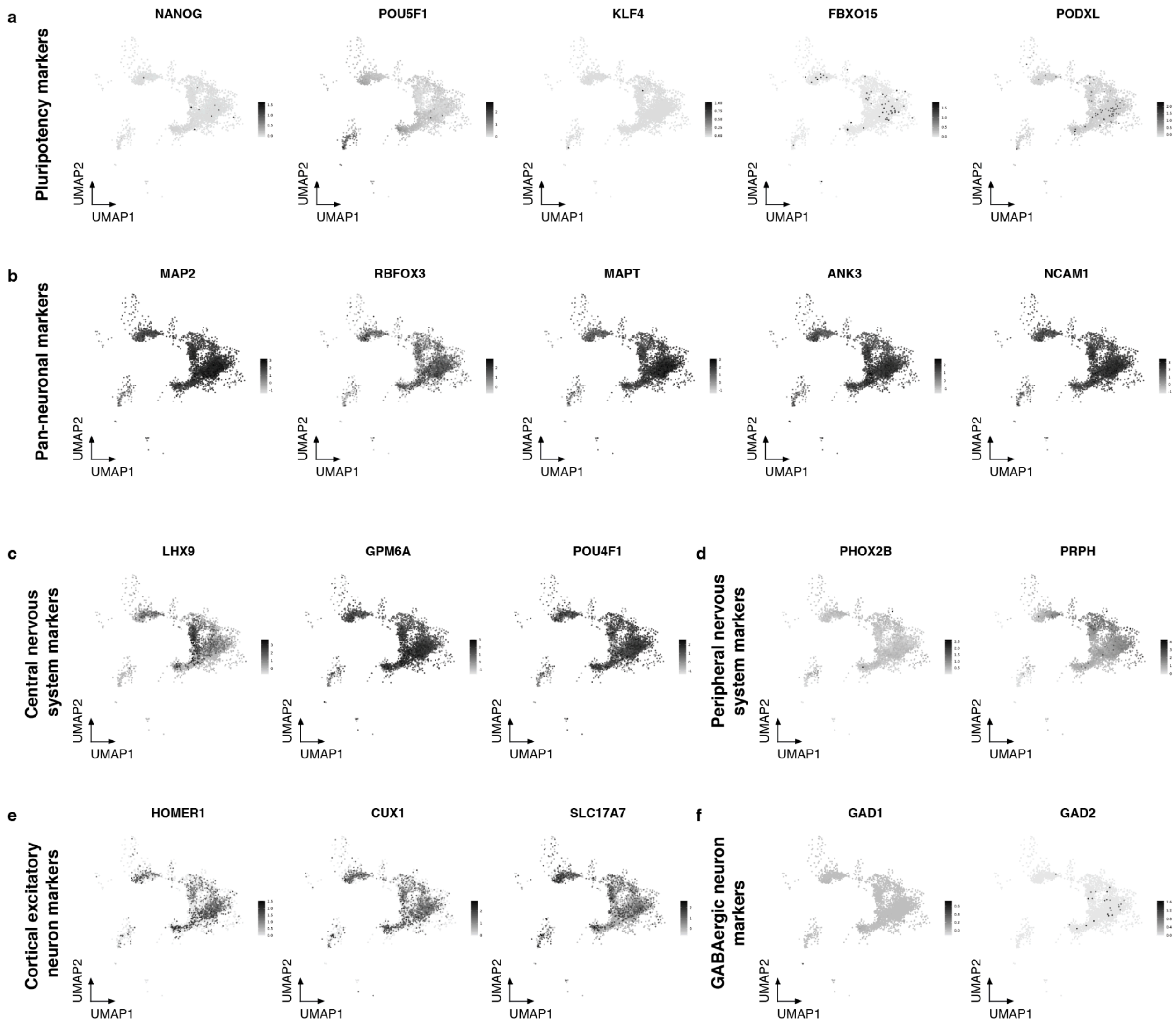

Supplementary Fig. 8 | Single-cell transcriptomic characterization of iPSC-derived neurons used in screen. a) Expression feature plots of canonical pluripotency markers *NANOG*, *POU5F1*, *KLF4*, *FBXO15*, and *PODXL*. **b)** Expression feature plots of pan-neuronal markers *MAP2*, *RBFOX3*, *MAPT*, *ANK3*, and *NCAM1*. **c)** Expression feature plots of central nervous system marker genes *LHX9*, *GPM6A*, and *POU4F1*. **d)** Expression feature plots of peripheral nervous system marker genes *PHOX2B* and *PRPH*. **e)** Expression feature plots of cortical excitatory neuron markers *HOMER1*, *CUX1*, and *SLC17A7*. **f)** Expression feature plots of GABAergic neuron marker genes *GAD1* and *GAD2*.

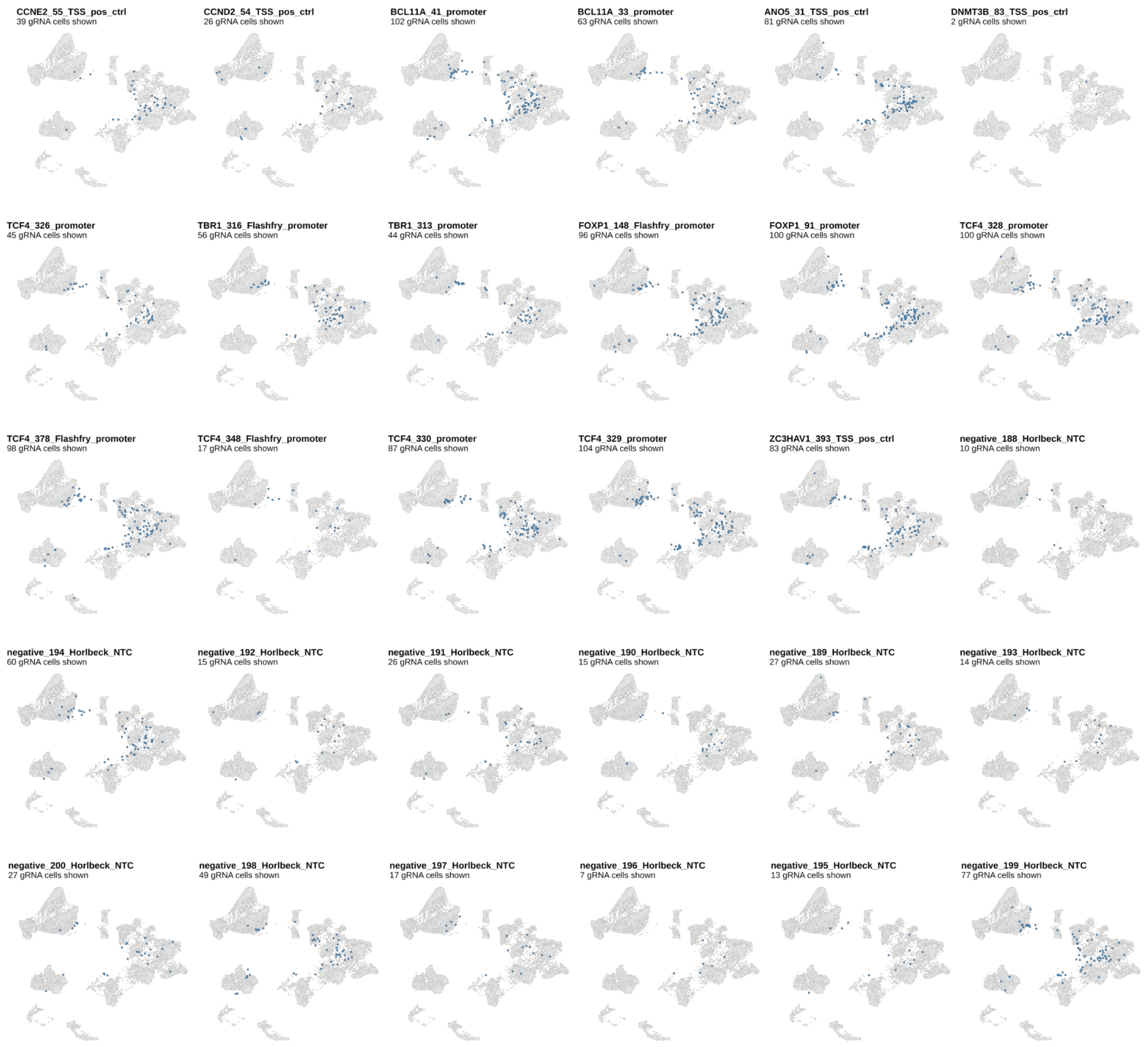

Supplementary Fig. 9 | Distribution of CRISPRa gRNAs in single-cell neuron transcriptome data. Cells harboring specific CRISPRa gRNAs (dark blue) overlaid onto *NGN2*-induced neuron differentiation transcriptome data[30]. No readily apparent spatial enrichment of gRNAs is observed in UMAP plots. Note that the CRISPRa dataset was randomly downsampled to 5000 cells for all UMAP comparison analyses.

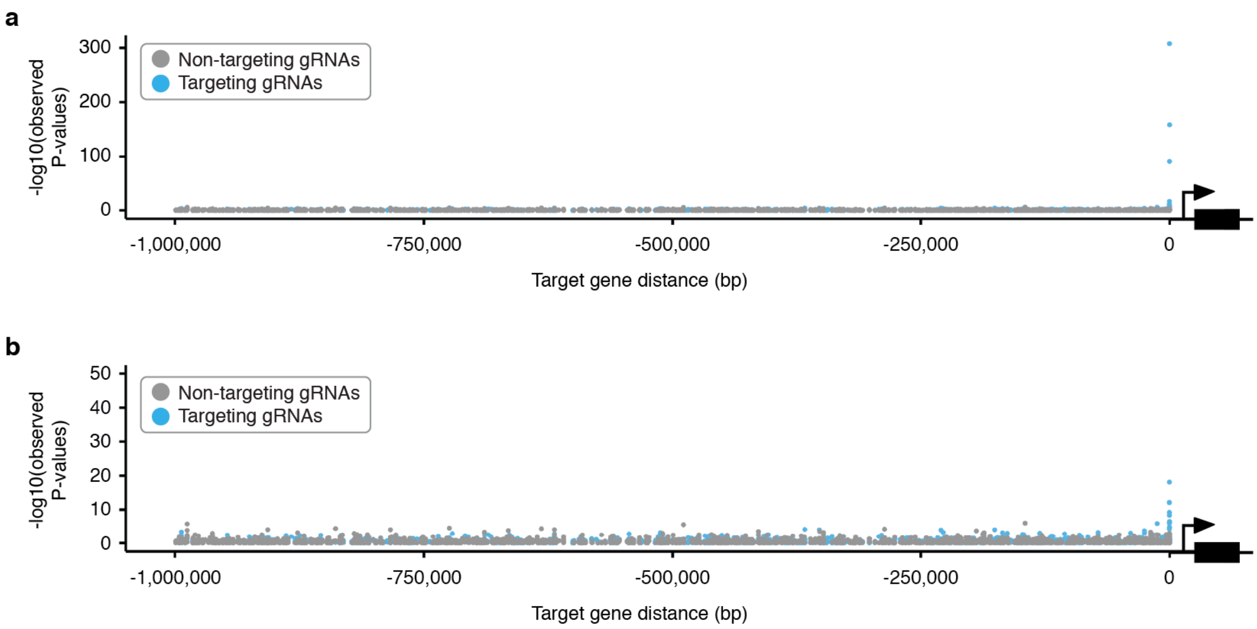

Supplementary Fig. 10 | Successful targeting gRNAs are enriched for genomic proximity to their paired target gene scores near target genes in the iPSC-derived neurons. a) Targeting gRNAs yielding upregulation are enriched for proximity to their target gene, while NTCs are not. **b)** Same plot as in **a**, with the y-axis clipped at 50. **a-b)** *P*-values are from a two-tailed Wilcoxon rank-sum test.

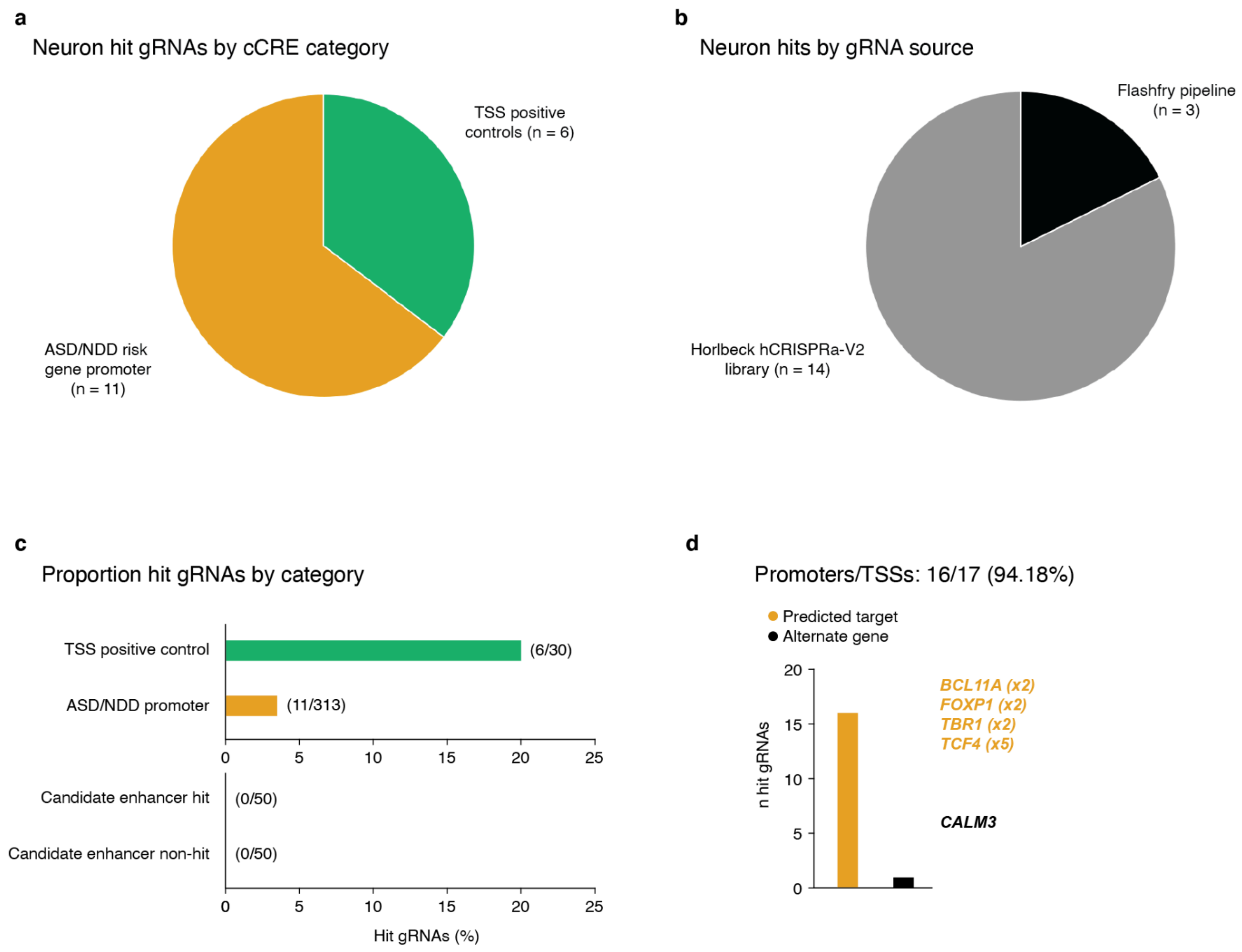

Supplementary Fig. 11 | Hit breakdown for screen conducted in iPSC-derived neurons. a) Neuron hit gRNAs by cCRE category. **b)** Neuron hit gRNAs by gRNA source library or design pipeline. **c)** Proportion of hit gRNAs by cCRE category. **d)** Proportion of hit gRNAs yielding upregulation of their intended/expected target gene or an alternate gene for candidate promoters/TSSs. Example hits targeting candidate NDD risk gene promoters are listed. Bracketed numbers denote the number of independent hit gRNAs targeting the same cCRE.

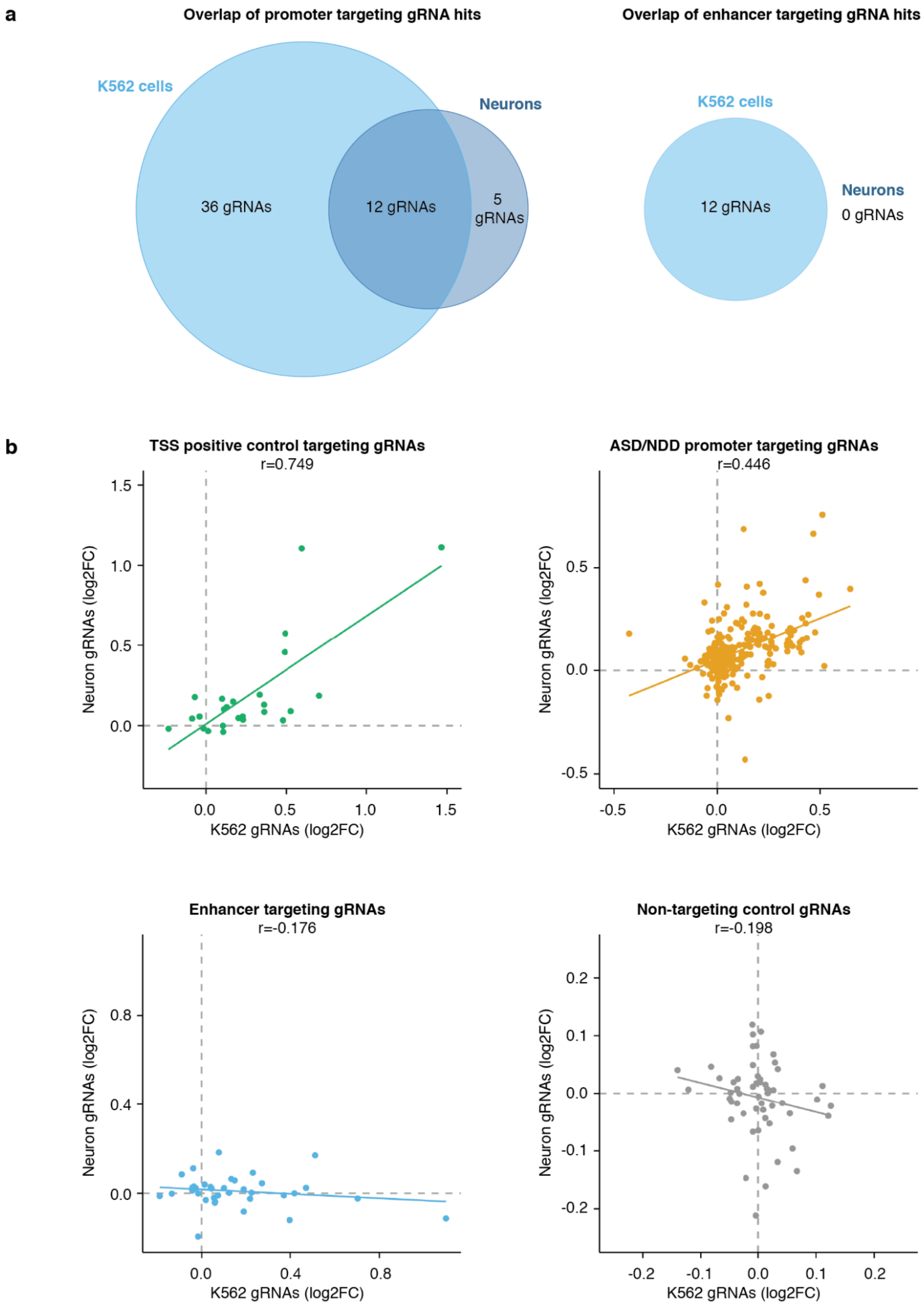

Supplementary Fig. 12 | Comparison of K562 vs. neuronal CRISPRa screening hits. a) Venn diagram showing number of overlapping promoter-targeting gRNA hits (left) and enhancer-targeting

gRNA hits (right) between the K562 and neuron CRISPRa screens. **b)** Correlation plots of log2 fold changes of TSS positive control targeting gRNAs (top left), ASD/NDD promoter targeting gRNAs (top right), enhancer targeting gRNAs (bottom left), and NTC gRNAs (bottom right) between the K562 and neuron CRISPRa screens.

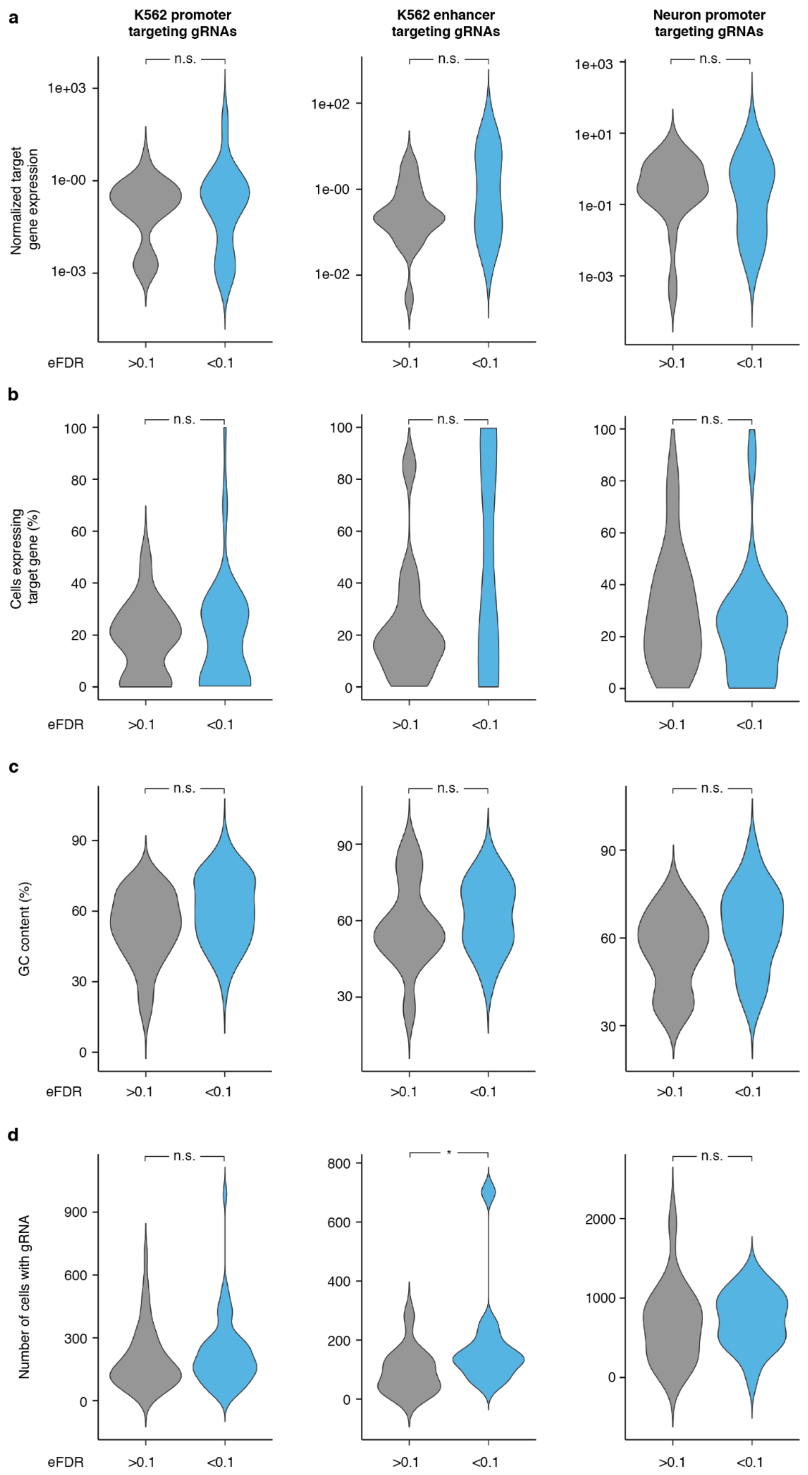

Supplementary Fig. 13 | Characteristics of gRNAs leading to upregulation at EFDR<0.1 vs. EFDR>0.1. a) Comparison of normalized gene expression values of targeted genes of gRNAs that resulted in an EFDR<0.1 (designated as “hit” gRNAs) versus gRNAs that resulted in an EFDR>0.1 (not designated as “hit” gRNAs). **b)** Comparison of the percentage of cells expressing the target gene of gRNAs that resulted in an EFDR<0.1 versus gRNAs that resulted in an EFDR>0.1. **c)** GC content (in

percent) of gRNAs that resulted in an EFDR<0.1 versus gRNAs that resulted in an EFDR>0.1. **d)** Number of cells harboring each gRNA for gRNAs that resulted in an EFDR<0.1 versus gRNAs that resulted in an EFDR>0.1. For all panels, K562 promoter-targeting gRNAs (left), K562 enhancer-targeting gRNAs (middle), and neuron promoter-targeting gRNAs (right) are shown. Abbreviations: n.s.: not significant (p>0.05, Wilcoxon rank-sum test), *: p<0.05 (Wilcoxon rank-sum test).

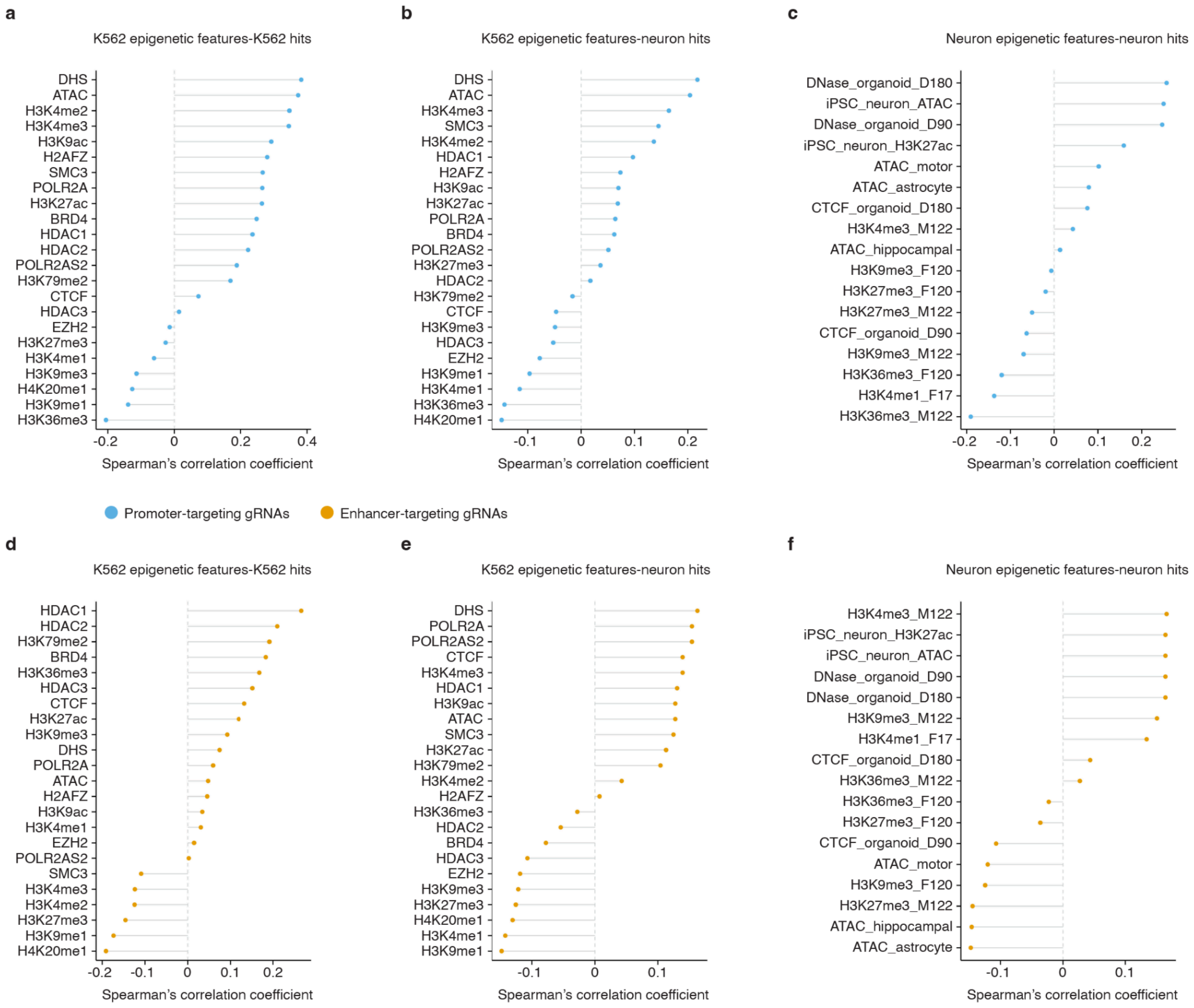

Supplementary Fig. 14 | Epigenetic features predictive of CRISPRa hit gRNAs. a) Correlation of K562 epigenetic features with hit vs. non-hit status for promoter-targeting K562 gRNAs. **b)** Correlation of K562 epigenetic features with hit vs. non-hit status for promoter-targeting neuron gRNAs. **c)** Correlation of brain tissue and organoid epigenetic features with hit vs. non-hit status for promoter-targeting neuron gRNAs. **d)** Correlation of K562 epigenetic features with hit vs. non-hit status for enhancer-targeting K562 gRNAs. **e)** Correlation of K562 epigenetic features with hit vs. non-hit status for enhancer-targeting neuron hit gRNAs. **f)** Correlation of brain tissue and organoid epigenetic features with hit vs. non-hit status for enhancer-targeting neuron hit gRNAs. Although neuron hits are correlated with K562 epigenetic features, these correlations are lower in magnitude than for K562 hits; this pattern may follow from overlap in accessible vs. repressed regions between the cell types, together with the deep extent to which K562 cells have been epigenetically profiled.

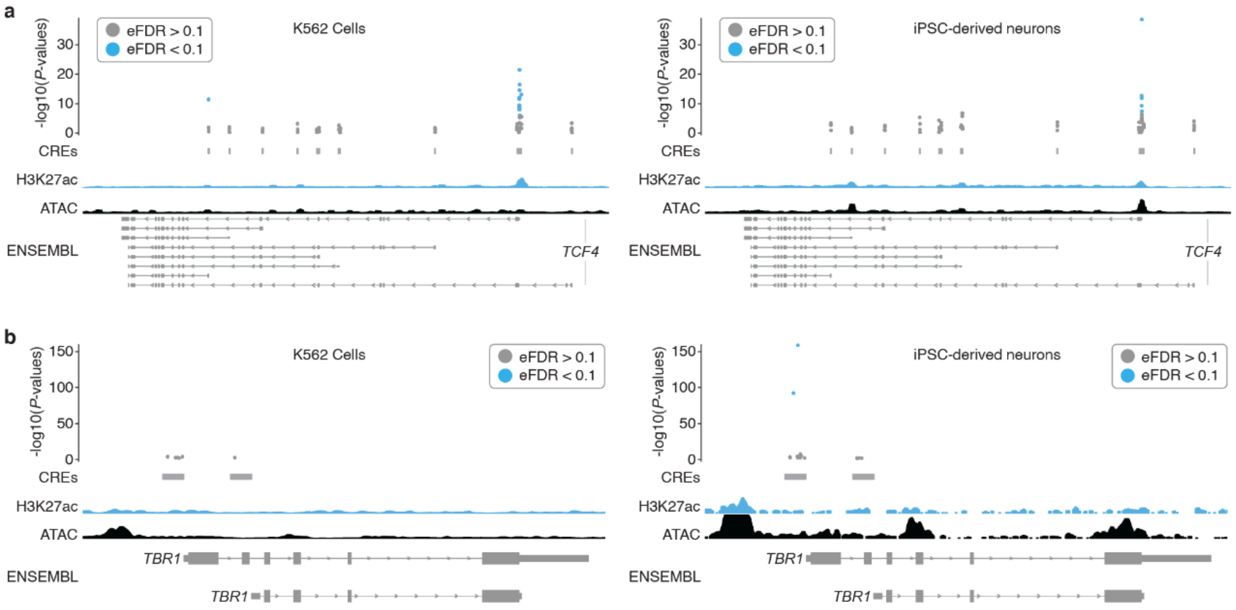

Supplementary Fig. 15 | TSS and cell-type specific promoters. a) The majority of hit gRNAs for *TCF4* target the same TSS in K562 cells and iPSC-derived neurons. *P*-values are visualized alongside tracks for K562 ATAC-seq (ENCODE), K562 H3K27ac signal (ENCODE), iPSC-derived neuron ATAC-seq (accessibility), iPSC-derived neuron H3K27ac and RefSeq validated transcripts (ENSEMBL/NCBI). *P*-values are from a two-tailed Wilcoxon rank-sum test. Coloured points indicate which perturbations drove significant upregulation using an Empirical FDR approach (EFDR < 0.1). **b)** Two hit gRNAs targeting the same TSS of *TBR1* drive upregulation specifically in iPSC-derived neurons. Genomic tracks are the same as in panel **a**.

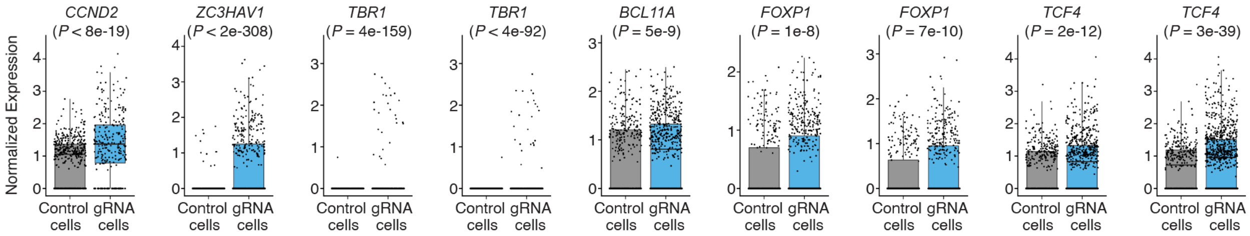

Supplementary Fig. 16 | Example upregulations in iPSC-derived neurons. Example violin plots showing the average log2 fold change between cells with a given gRNA and controls for select hit gRNAs. Hits include TSS positive controls (*CCND2*, *ZC3HAV1*), candidate promoters of genes rarely or not expressed NGN2, including the cortical neuron marker *TBR1*, and candidate promoters of genes with native expression in iPSC-derived neurons that could be further upregulated (*BCL11A*, *FOXP1*, and *TCF4*). Number of cells bearing each targeting gRNA (from left to right): *CCND2* (n = 350), *ZC3HAV1* (n = 765), *TBR1* (n = 455), *TBR1* (n = 655), *BCL11A* (n = 964), *FOXP1* (n = 1198), *FOXP1* (n = 917), *TCF4* (n = 1051), *TCF4* (n = 1253). Control cells are downsampled to have the same number of cells as the average number of cells detected per gRNA (n = 638) for visualization. Boxes represent the 25th, 50th, and 75th percentiles. Whiskers extend from hinge to 1.5 times the inter-quartile range. All data points are also plotted on top of the box plot for transparency. *P*-values are from a two-tailed Wilcoxon rank-sum test. Normalized expression values represent log normalized expression values from Seurat.

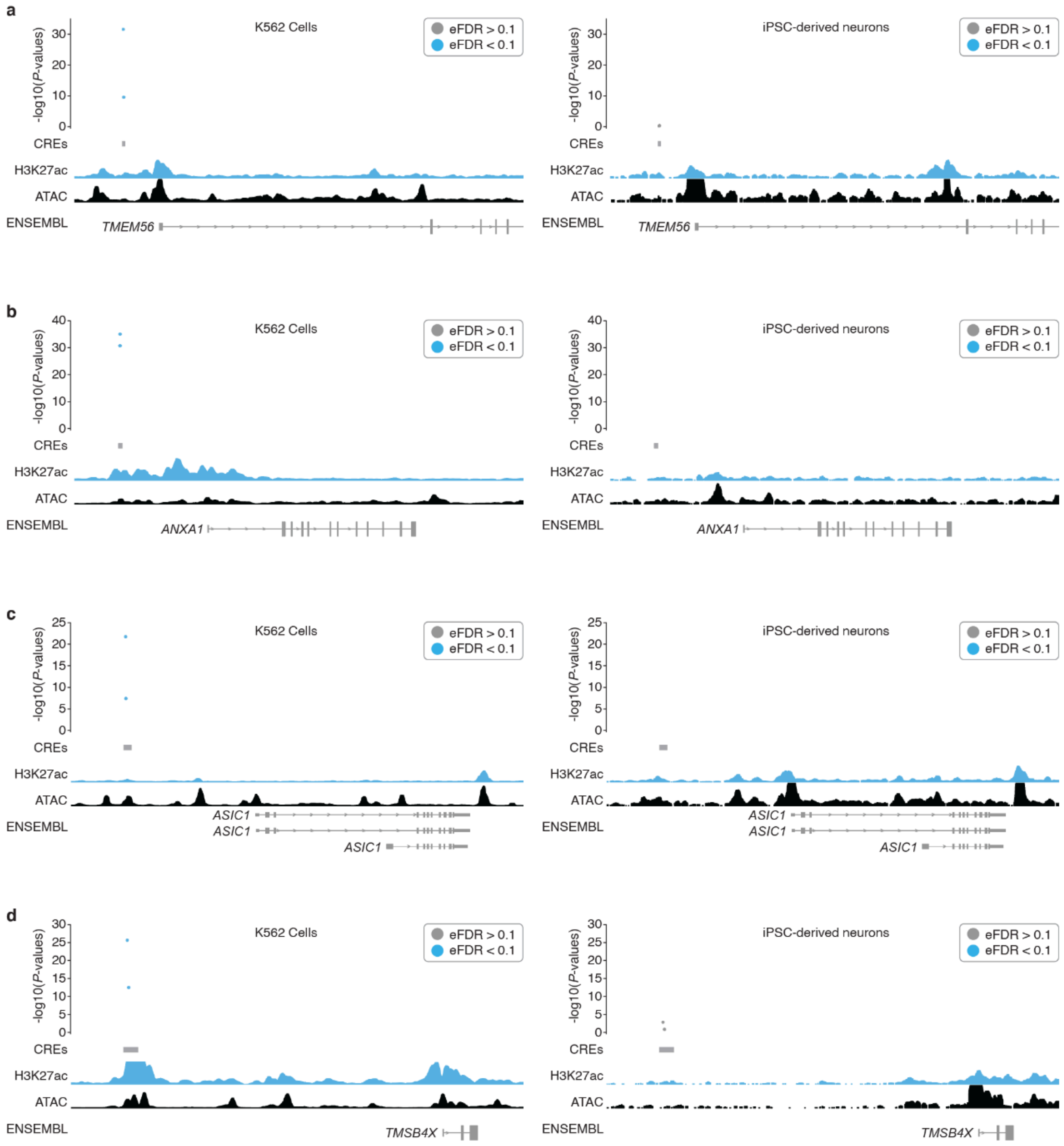

Supplementary Fig. 17 | Cell-type specific enhancers. a-d) *P*-values are visualized alongside tracks for K562 ATAC-seq (ENCODE), K562 H3K27ac signal (ENCODE), iPSC-derived neuron ATAC-seq (accessibility), iPSC-derived neuron H3K27ac and RefSeq validated transcripts (ENSEMBL/NCBI). *P*-values are from a two-tailed Wilcoxon rank-sum test. Coloured points indicate which perturbations drove significant upregulation using an Empirical FDR approach (EFDR < 0.1). All K562 enhancer hits were cell type specific. Enhancers with multiple hit gRNAs are shown.

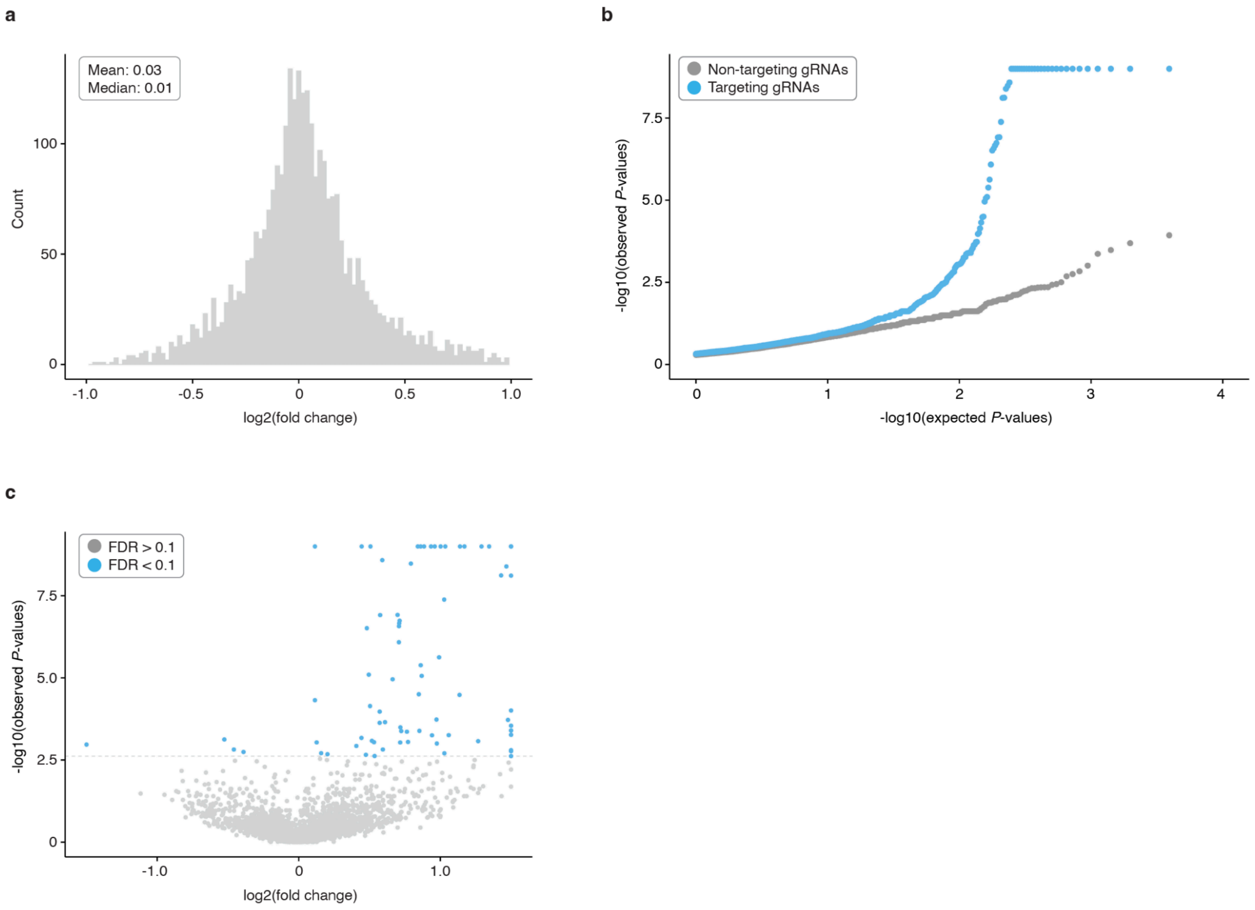

Supplementary Fig. 18 | SCEPTRE results for K562 cells. a) Log2 fold change distribution for NTC gRNA tests in SCEPTRE framework. A NTC distribution centered at zero is expected for well-calibrated screens. **b)** QQ-plot showing the distribution of expected vs. observed *P*-values for targeting (blue) and non-targeting (gray, downsampled) differential expression tests from SCEPTRE. There is an excess of highly significant *P*-values in targeting tests compared to controls. **c)** Volcano plot showing the average log2 fold change and *P*-values for targeting tests. **b-c)** Two-tailed SCEPTRE *P*-values were derived using default parameters. SCEPTRE *P*-values were then Benjamini-Hochberg corrected and those < 0.1 were kept for two-sided discovery sets.

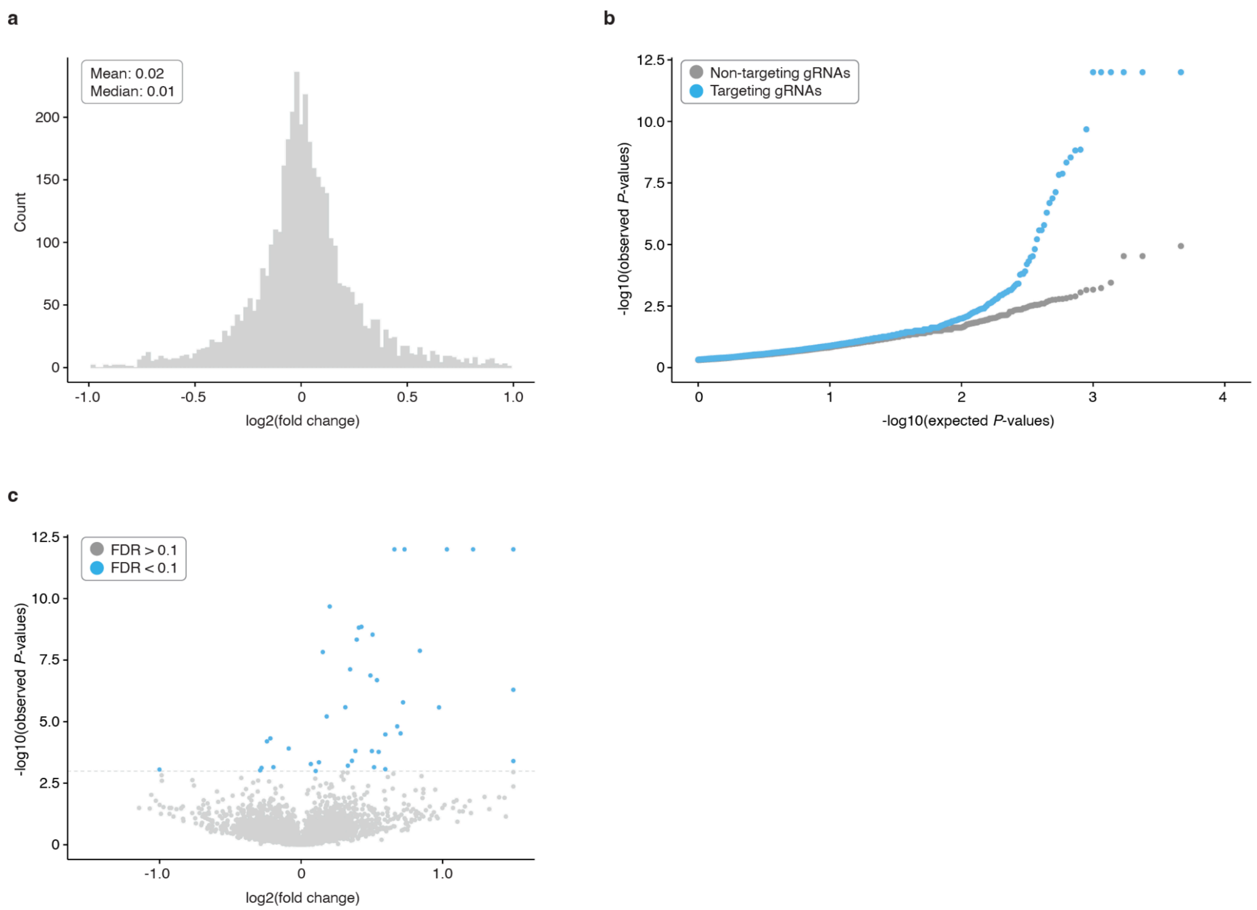

Supplementary Fig. 19 | SCEPTRE results for iPSC-derived neurons. a) Log2 fold change distribution for NTC gRNA tests in SCEPTRE framework. A NTC distribution centered at zero is expected for well-calibrated screens. **b)** QQ-plot showing the distribution of expected vs. observed *P*-values for targeting (blue) and non-targeting (gray, downsampled) differential expression tests from SCEPTRE. There is an excess of highly significant *P*-values in targeting tests compared to controls. **c)** Volcano plot showing the average log2 fold change and *P*-values for targeting tests. **b-c)** Two-tailed SCEPTRE *P*-values were derived using default parameters. SCEPTRE *P*-values were then Benjamini-Hochberg corrected and those < 0.1 were kept for two-sided discovery sets.

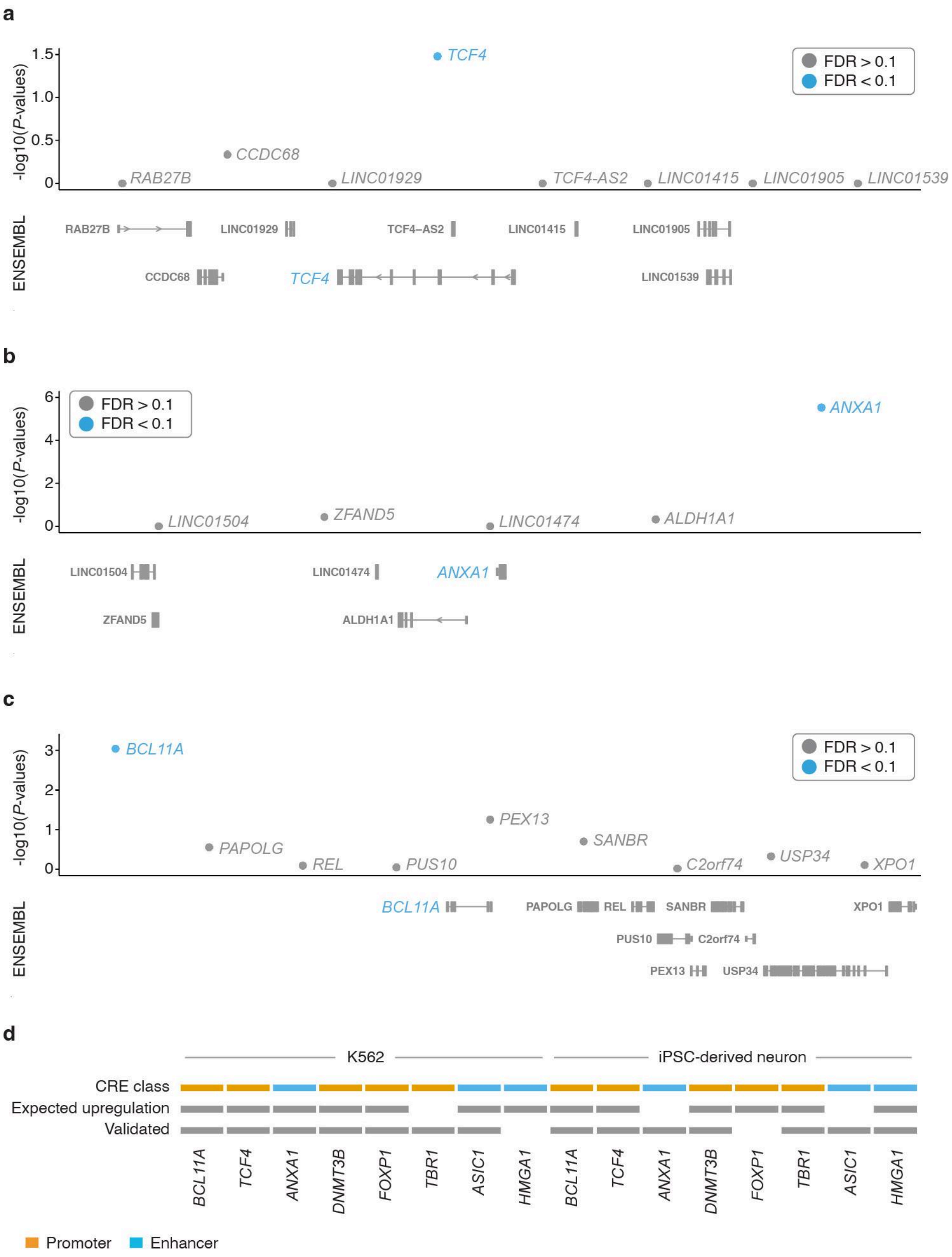

Supplementary Fig. 20 | Singleton validations confirm target upregulations. a) *TCF4* upregulation in K562 cells expressing a *TCF4* promoter-targeting gRNA compared to control cells. *P*-values (DESeq2) from differential expression tests of the intended target (blue) are visualized alongside neighboring genes within 1Mb of the target site (gray). Neighboring genes are filtered to those detected in the sc-RNA-seq data to aid visualization. **b)** *ANXA1* upregulation in K562 cells expressing an *e-ANXA1* enhancer-targeting gRNA compared to control cells. Genomic tracks are the same as in panel **a**. **c)** *BCL11A* upregulation in iPSC-derived neurons expressing an *BCL11A* promoter-targeting gRNA compared to control cells. Genomic tracks are the same as in panel **a**. **d)** Categorical heatmap indicating whether a given validation

gRNA targeted a promoter or enhancer, whether the gRNA was expected to drive upregulation in that cell type based on the multiplex screen, and whether the expected upregulation (or not) was observed in singleton validations.

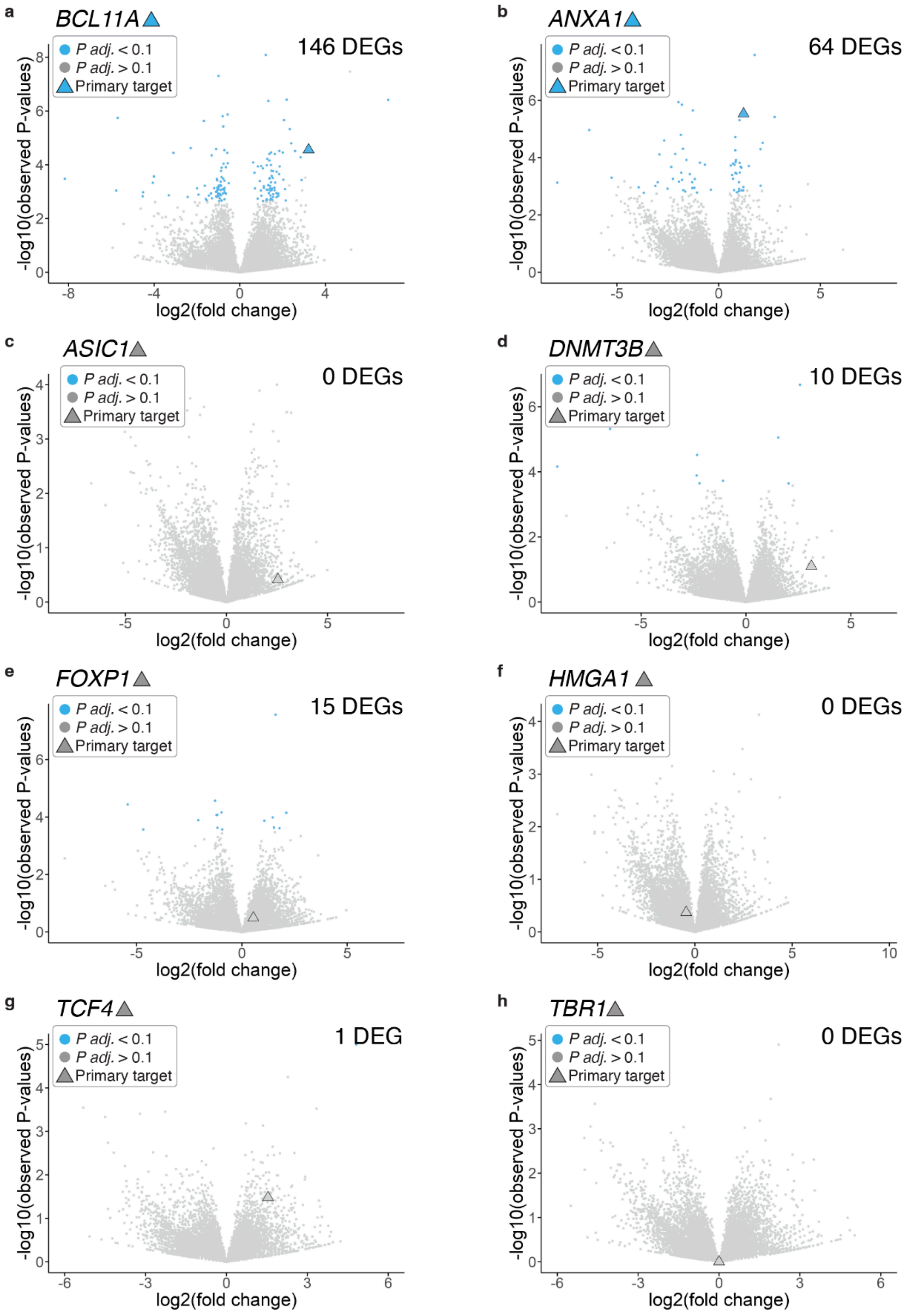

Supplementary Fig. 21 | Genome-wide differential expression analysis of singleton validations in K562 cells. a) Genome-wide differential gene expression analysis comparing cell lines harboring a *BCL11A* promoter-targeting gRNA compared to all other lines as a control. **b-h)** Differential gene expression analyses as in panel **a)** for gRNAs targeting cCREs of: *ANXA1* (**b**), *ASIC1* (**c**), *DNMT3B* (**d**), *FOXP1* (**e**), HMGA1 (**f**), TCF4 (**g**), and TBR1 (**h**). Differential expression *P*-values and number of Differentially Expressed Genes (DEGs) from DESeq2.

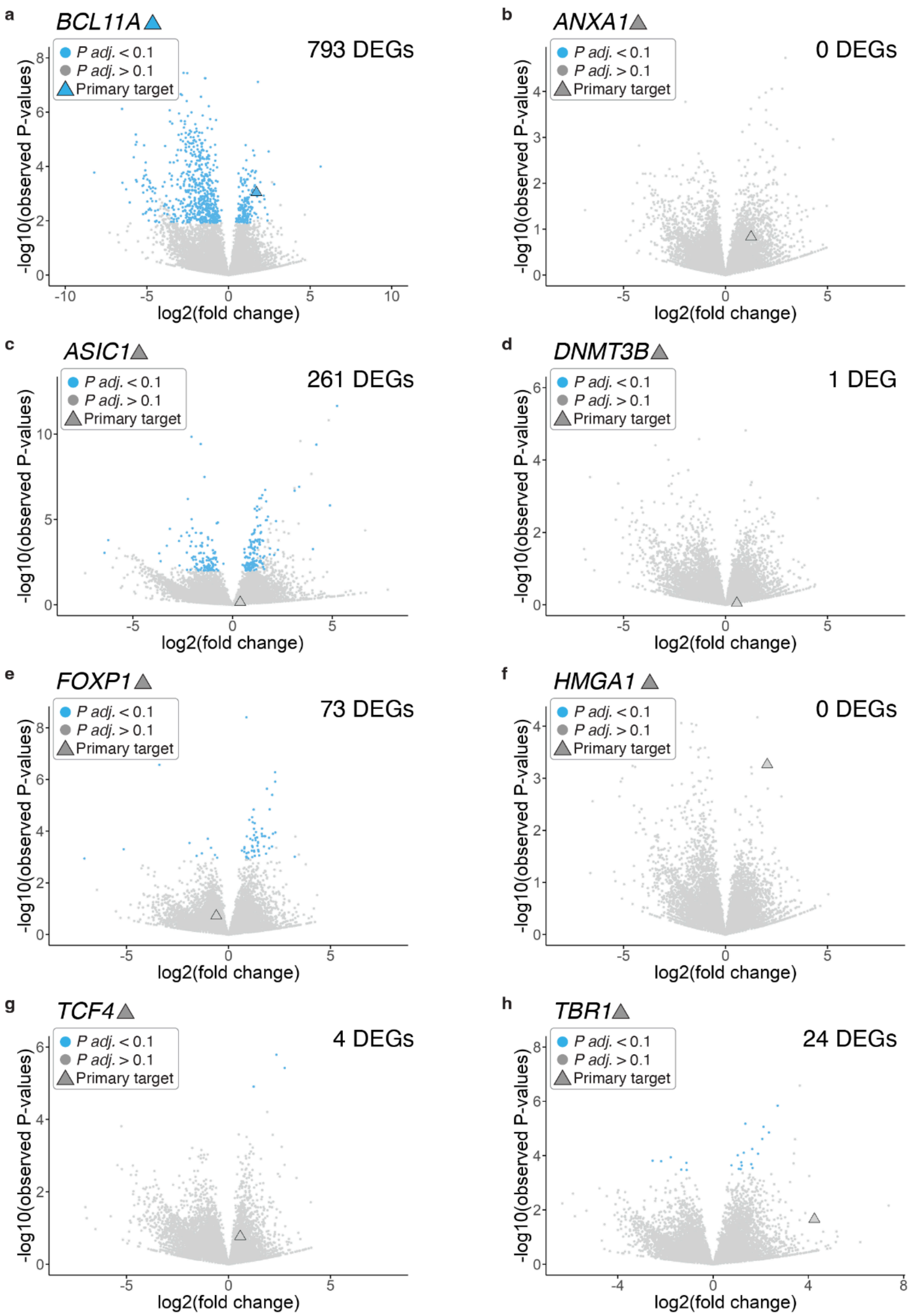

Supplementary Fig. 22 | Genome-wide differential expression analysis of singleton validations in iPSC-derived neurons. a) Genome-wide differential gene expression analysis comparing cell lines harboring a *BCL11A* promoter-targeting gRNA compared to all other lines as a control. **b-h)** Differential gene expression analyses as in panel **a)** for gRNAs targeting cCREs of: *ANXA1* (**b**), *ASIC1* (**c**), *DNMT3B* (**d**), *FOXP1* (**e**), HMGA1 (**f**), TCF4 (**g**), and TBR1 (**h**). Differential expression *P*-values and number of Differentially Expressed Genes (DEGs) from DESeq2.

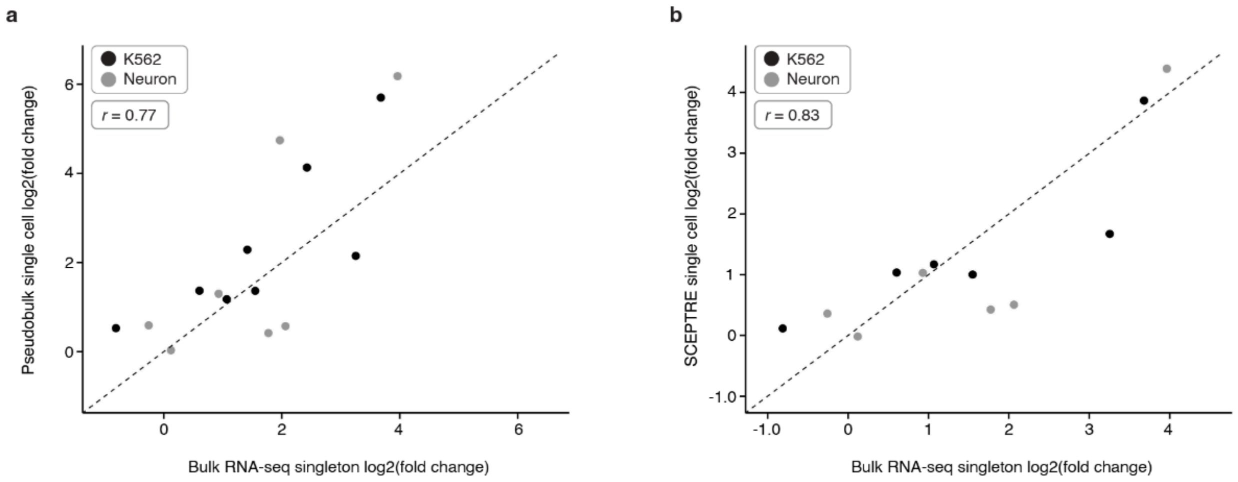

Supplementary Fig. 23 | Correlation of fold change estimates from singleton bulk RNA-seq and multiplex single cell CRISPRa experiments. a) Correlation of log2 fold change estimates from singleton bulk RNA-seq CRISPRa experiments and pseudobulked single cells from the multiplex single cell CRISPRa experiment. Note that TBR1 expression was not detected in K562 cells precluding calculation of fold changes. Points are coloured according to cell context (K562 or iPSC-derived neurons). **b)** Correlation of log2 fold change estimates from singleton bulk RNA-seq CRISPRa experiments and SCEPTRE log2 fold change estimates from the multiplex single cell CRISPRa experiment. Note that *TBR1* and *ASIC1* in K562, and *DNMT3B* and *ANXA1* in iPSC-derived neurons, did not meet SCEPTRE's minimum threshold for proportion of cells expressing a target gene, precluding calculation of fold changes. Points are coloured according to cell context (K562 or iPSC-derived neurons). Modestly increased correlation between SCEPTRE and bulk log2 fold change estimates is potentially due to accounting for various technical factors (sequencing depth, cell quality, etc.) that direct pseudobulking does not. Pearson's correlation coefficient is shown.
