# Supplementary material for: Multiplex, single-cell CRISPRa screening for cell type specific regulatory elements
Source: Nat Commun. 2024 Sep 18;15:8209. doi: 10.1038/s41467-024-52490-4 (PMC11411074; doi:10.1038/s41467-024-52490-4)
Supplement: Supplementary file 3 — Description of Additional Supplementary Files [file 41467_2024_52490_MOESM3_ESM.pdf]

Description of Additional Supplementary Files

File Name: Supplementary Data 1

Description: gRNA sequences.

File Name: Supplementary Data 2

Description: K562 full screen results. P-values are from a two-tailed Wilcoxon rank-sum test.

File Name: Supplementary Data 3

Description: K562 primary target results. P-values are from a two-tailed Wilcoxon rank-sum test.

File Name: Supplementary Data 4

Description: K562 hits (EFDR < 0.1). P-values are from a two-tailed Wilcoxon rank-sum test. EFDR < 0.1 sets were defined as described in the methods section.

File Name: Supplementary Data 5

Description: iPSC-derived neuron full screen results. P-values are from a two-tailed Wilcoxon rank-sum test.

File Name: Supplementary Data 6

Description: iPSC-derived neuron primary target results P-values are from a two-tailed Wilcoxon rank-sum test.

File Name: Supplementary Data 7

Description: iPSC-derived neuron hits (EFDR < 0.1). P-values are from a two-tailed Wilcoxon rank-sum test. EFDR < 0.1 sets were defined as described in the methods section.

File Name: Supplementary Data 8

Description: Epigenetic feature datasets.

File Name: Supplementary Data 9

Description: SCEPTRE K562 NTC calibration results. Two-tailed SCEPTRE P-values were derived using default parameters.

File Name: Supplementary Data 10

Description: SCEPTRE K562 targeting discovery results. Two-tailed SCEPTRE P-values were derived using default parameters.

File Name: Supplementary Data 11

Description: SCEPTRE K562 hits (FDR < 0.1). Two-tailed SCEPTRE P-values were derived using default parameters. SCEPTRE P-values were then Benjamini-Hochberg corrected and those < 0.1 were kept for two-sided discovery sets.

File Name: Supplementary Data 12

Description: SCEPTRE iPSC-derived neuron NTC calibration results. Two-tailed SCEPTRE P-values were derived using default parameters.

File Name: Supplementary Data 13

Description: SCEPTRE iPSC-derived neuron targeting discovery results. Two-tailed SCEPTRE P-values were derived using default parameters.

File Name: Supplementary Data 14

Description: SCEPTRE iPSC-derived neuron hits (FDR < 0.1). Two-tailed SCEPTRE P-values were derived using default parameters. SCEPTRE P-values were then Benjamini-Hochberg corrected and those < 0.1 were kept for two-sided discovery sets.

File Name: Supplementary Data 15

Description: gRNA set for singleton validations.

File Name: Supplementary Data 16

Description: Singleton validation differentially expressed genes.
