# Supplementary material for: Multiplex, single-cell CRISPRa screening for cell type specific regulatory elements
Source: Nat Commun. 2024 Sep 18;15:8209. doi: 10.1038/s41467-024-52490-4 (PMC11411074; doi:10.1038/s41467-024-52490-4)
Supplement: Supplementary file 20 — Reporting Summary [file 41467_2024_52490_MOESM20_ESM.pdf]

nature portfolio

Corresponding author(s): Jay Shendure and Nadav Ahituv

Last updated by author(s): Aug 8, 2024

Reporting Summary

Nature Portfolio wishes to improve the reproducibility of the work that we publish. This form provides structure for consistency and transparency in reporting. For further information on Nature Portfolio policies, see our Editorial Policies and the Editorial Policy Checklist.

Statistics

For all statistical analyses, confirm that the following items are present in the figure legend, table legend, main text, or Methods section.

n/a	Confirmed	
☐	☒	The exact sample size (*n*) for each experimental group/condition, given as a discrete number and unit of measurement
☐	☒	A statement on whether measurements were taken from distinct samples or whether the same sample was measured repeatedly
☐	☒	The statistical test(s) used AND whether they are one- or two-sided<br>*Only common tests should be described solely by name; describe more complex techniques in the Methods section.*
☐	☒	A description of all covariates tested
☐	☒	A description of any assumptions or corrections, such as tests of normality and adjustment for multiple comparisons
☐	☒	A full description of the statistical parameters including central tendency (e.g. means) or other basic estimates (e.g. regression coefficient) AND variation (e.g. standard deviation) or associated estimates of uncertainty (e.g. confidence intervals)
☐	☒	For null hypothesis testing, the test statistic (e.g. F, t, r) with confidence intervals, effect sizes, degrees of freedom and P value noted<br>*Give P values as exact values whenever suitable.*
☒	☐	For Bayesian analysis, information on the choice of priors and Markov chain Monte Carlo settings
☒	☐	For hierarchical and complex designs, identification of the appropriate level for tests and full reporting of outcomes
☐	☒	Estimates of effect sizes (e.g. Cohen's *d*, Pearson's *r*), indicating how they were calculated

Our web collection on statistics for biologists contains articles on many of the points above.

Software and code

Policy information about availability of computer code

Data collection	No software was used for data collection.
Data analysis	All code and scripts used for analyses are all publicly available and are accessible on Github via the following link [https://github.com/shendurelab/multiplex_scCRISPRa_screening] or at [https://krishna.gs.washington.edu/content/members/CRISPRa_QTL_website/public/].

For manuscripts utilizing custom algorithms or software that are central to the research but not yet described in published literature, software must be made available to editors and reviewers. We strongly encourage code deposition in a community repository (e.g. GitHub). See the Nature Portfolio guidelines for submitting code & software for further information.

Data

Policy information about availability of data

All manuscripts must include a data availability statement. This statement should provide the following information, where applicable:

- Accession codes, unique identifiers, or web links for publicly available datasets
- A description of any restrictions on data availability
- For clinical datasets or third party data, please ensure that the statement adheres to our policy

The raw data, processed data, and corresponding metadata are have been deposited to the IGVF database under accession codes IGVFDS9078ZWQH [https://data.igvf.org/analysis-sets/IGVFDS9078ZWQH/] and IGVFDS4021XJLW [https://data.igvf.org/analysis-sets/IGVFDS4021XJLW/]. These are also available at[https://krishna.gs.washington.edu/content/members/CRISPRa_QTL_website/public/]. Published data used: GSE170378[https://www.ncbi.nlm.nih.gov/geo/query/acc.cgi?

acc=GSE170378], GSM733656[https://www.ncbi.nlm.nih.gov/geo/query/acc.cgi?acc=GSM733656], GSE113483[https://www.ncbi.nlm.nih.gov/geo/query/acc.cgi?acc=GSE113483]. Source data are provided with this paper.

Research involving human participants, their data, or biological material

Policy information about studies with human participants or human data. See also policy information about sex, gender (identity/presentation), and sexual orientation and race, ethnicity and racism.

Reporting on sex and gender	n/a
Reporting on race, ethnicity, or other socially relevant groupings	n/a
Population characteristics	n/a
Recruitment	n/a
Ethics oversight	n/a

Note that full information on the approval of the study protocol must also be provided in the manuscript.

Field-specific reporting

Please select the one below that is the best fit for your research. If you are not sure, read the appropriate sections before making your selection.

☒ Life sciences ☐ Behavioural & social sciences ☐ Ecological, evolutionary & environmental sciences

For a reference copy of the document with all sections, see nature.com/documents/nr-reporting-summary-flat.pdf

Life sciences study design

All studies must disclose on these points even when the disclosure is negative.

Sample size	No sample size calculation was performed. Multiplex, single-cell CRISPRa screen scale was designed to meet or exceed standards in the field.
Data exclusions	No data exclusions.
Replication	Multiplex single-cell CRISPR screens were analyzed in aggregate (without biological replicates). Count matrices and associated metadata were used to construct a single cell covariate matrix with gRNA and gene expression library size (total UMIs), unique genes (non-zero expression in gRNA and gene expression libraries), cell line (in the case of K562 cells with differing CRISPRa effectors), whether cells were GFP sorted prior to profiling, 10x lane, and percent mitochondrial reads input as covariates during model fitting. Multiplex screen scores for a subset of sgRNAs were confirmed to correspond with individual validation experiments in both cell contexts.
Randomization	n/a
Blinding	n/a

Reporting for specific materials, systems and methods

We require information from authors about some types of materials, experimental systems and methods used in many studies. Here, indicate whether each material, system or method listed is relevant to your study. If you are not sure if a list item applies to your research, read the appropriate section before selecting a response.

Materials & experimental systems

n/a	Involved in the study	
☒	☐	Antibodies
☒	☐	Eukaryotic cell lines
☒	☐	Palaeontology and archaeology
☒	☐	Animals and other organisms
☒	☐	Clinical data
☒	☐	Dual use research of concern
☒	☐	Plants

Methods

n/a	Involved in the study	
☒	☐	ChIP-seq
☐	☒	Flow cytometry
☒	☐	MRI-based neuroimaging

Plants

Seed stocks	n/a
Novel plant genotypes	n/a
Authentication	n/a

Flow Cytometry

Plots

Confirm that:

☒ The axis labels state the marker and fluorochrome used (e.g. CD4-FITC).

☒ The axis scales are clearly visible. Include numbers along axes only for bottom left plot of group (a 'group' is an analysis of identical markers).

☒ All plots are contour plots with outliers or pseudocolor plots.

☒ A numerical value for number of cells or percentage (with statistics) is provided.

Methodology

Sample preparation	iPSC-derived neurons were prepared for sorting as described in the Methods section (in "10x Genomics sc-RNA-seq with associated gRNA transcript capture" and subsection "iPSC-derived neuron screen").
Instrument	BD FACSAria II
Software	FlowJo software was used to analyze the data.
Cell population abundance	The FACS analysis that was done in Supplementary Figure 2 was purely an analysis (no sorting performed). The GFP+ sorting that was done for the neuron single-cell preparation is described in the Methods section (in "10x Genomics sc-RNA-seq with associated gRNA transcript capture" and subsection "iPSC-derived neuron screen").
Gating strategy	Gating was performed by determining the fluorescence of the negative control, and gating such that 0% of the negative control had fluorescent cells.

☒ Tick this box to confirm that a figure exemplifying the gating strategy is provided in the Supplementary Information.
